# Adult inactivation of the *Reln* gene leads to disruption of cortical lamination and rhythmic activity

**DOI:** 10.1093/pnasnexus/pgag363

**Published:** 2026-10-29

**Authors:** Yasmina Manso, Nuria Masachs, Alba Vílchez-Acosta, Victoria González Sabater, Gerardo Contreras-Murillo, Raudel Sánchez-Campusano, Marc Hernaiz-Llorens, Alba Elias-Tersa, María Ángeles Llanos, Gerard Rovira-Escalé, Ana Guerrero, Magdalena Martínez-Losa, Manuel Álvarez-Dolado, José María Delgado-García, Angus C Nairn, Agnès Gruart, Eduardo Soriano

**Affiliations:** Department of Cell Biology, Physiology and Immunology, Faculty of Biology and Institute of Neurosciences, University of Barcelona, Barcelona 08028, Spain; Centro de Investigación Biomédica en Red Enfermedades Neurodegenerativas (CIBERNED), ISCIII, Madrid 28031, Spain; Department of Cell Biology, Physiology and Immunology, Faculty of Biology and Institute of Neurosciences, University of Barcelona, Barcelona 08028, Spain; Department of Cell Biology, Physiology and Immunology, Faculty of Biology and Institute of Neurosciences, University of Barcelona, Barcelona 08028, Spain; Centro de Investigación Biomédica en Red Enfermedades Neurodegenerativas (CIBERNED), ISCIII, Madrid 28031, Spain; Department of Cell Biology, Physiology and Immunology, Faculty of Biology and Institute of Neurosciences, University of Barcelona, Barcelona 08028, Spain; Division of Neurosciences, Pablo de Olavide University, Sevilla 41013, Spain; Division of Neurosciences, Pablo de Olavide University, Sevilla 41013, Spain; Department of Cell Biology, Physiology and Immunology, Faculty of Biology and Institute of Neurosciences, University of Barcelona, Barcelona 08028, Spain; Department of Cell Biology, Physiology and Immunology, Faculty of Biology and Institute of Neurosciences, University of Barcelona, Barcelona 08028, Spain; Centro de Investigación Biomédica en Red Enfermedades Neurodegenerativas (CIBERNED), ISCIII, Madrid 28031, Spain; Department of Cell Biology, Physiology and Immunology, Faculty of Biology and Institute of Neurosciences, University of Barcelona, Barcelona 08028, Spain; Department of Cell Biology, Physiology and Immunology, Faculty of Biology and Institute of Neurosciences, University of Barcelona, Barcelona 08028, Spain; Department of Cell Biology, Physiology and Immunology, Faculty of Biology and Institute of Neurosciences, University of Barcelona, Barcelona 08028, Spain; Centro de Investigación Biomédica en Red Enfermedades Neurodegenerativas (CIBERNED), ISCIII, Madrid 28031, Spain; Andalusian Center for Molecular Biology and Regenerative Medicine-CABIMER, CSIC, Seville 41092, Spain; Andalusian Center for Molecular Biology and Regenerative Medicine-CABIMER, CSIC, Seville 41092, Spain; Division of Neurosciences, Pablo de Olavide University, Sevilla 41013, Spain; Department of Psychiatry, Yale University School of Medicine, New Haven, CT 06508, USA; Division of Neurosciences, Pablo de Olavide University, Sevilla 41013, Spain; Department of Cell Biology, Physiology and Immunology, Faculty of Biology and Institute of Neurosciences, University of Barcelona, Barcelona 08028, Spain; Centro de Investigación Biomédica en Red Enfermedades Neurodegenerativas (CIBERNED), ISCIII, Madrid 28031, Spain

**Keywords:** Reelin, cortical layering, LTP, psychiatric diseases, adult plasticity, rhythmic activity

## Abstract

Cortical cytoarchitecture and lamination are believed to remain stable after completion of development. Here, we demonstrate that early postnatal and adult (P45–P60) inactivation of the *Reln* gene results in dispersion of layers II and III neocortical neurons which invade layer I. In addition, neuronal mispositioning was observed in the dentate gyrus, the CA1–CA3 regions and in the cerebellum. Interestingly, inactivation of the *Reln* gene at adult stages resulted in altered oscillatory network activity in different cortical regions and in the nucleus accumbens. Moreover, Reelin deficiency increased depression-like behavior and impaired long-term memory consolidation. Thus, besides its crucial developmental role, Reelin is important in maintaining adult cortical lamination and rhythmic neuronal synchronization. These results suggest that, in addition to physiological and synaptic plasticity, there may be a certain degree of laminar structural plasticity in adult stages, which may lead to reconsidering the origin of layering alterations observed in neuropsychiatric diseases.

Significance Statement We demonstrate that deletion of a single gene (*Reln*) in the adult brain causes neuronal dispersion in different regions of the cerebral cortex and cerebellum. This occurs concomitantly with a disruption of rhythmic oscillatory activity. Thus, a certain degree of laminar plasticity may persist in the adult brain. Given the role of Reelin in the pathogenesis of psychiatric and neurodegenerative diseases, our data suggest a role for Reelin in the alteration of rhythms typical of these disorders.

## Introduction

The extracellular protein Reelin is crucial for brain development, including neuronal migration, synaptogenesis, and synaptic maturation (1–5). In the adult brain, Reelin is expressed by GABAergic interneurons in the cerebral cortex (6) and regulates glutamatergic receptor composition and neurotransmission, dendritic spine dynamics, long-term potentiation (LTP), and adult neurogenesis (7–11). Reelin deficits are associated with numerous neuropsychiatric and neurodegenerative diseases (12–15), indicating that Reelin is a key homeostatic regulator of different plasticity and neurotransmission processes in the adult brain.

Studies that evaluate Reelin function in the adult brain are hampered by the developmental defects in brain lamination in Reelin-deficient (*Reeler*) mice and *Reeler*-like mice, raising the question as to whether the defects detected during adulthood might be secondary to neuronal mispositioning during development. Heterozygous reeler mice do not appear to show neuroanatomical changes and have subtle behavioral and physiological alterations (16, 17). Although Reelin controls the migration of adult-born neurons into neurogenic niches such as the dentate gyrus and subventricular zone (8, 18, 19) a role for Reelin in adult brain structuring and layering has not been described; however, granule cell dispersion in the dentate gyrus in epileptic-like conditions has been linked to reduced Reelin protein levels (20, 21). To investigate the role of Reelin in postnatal and adult stages (bypassing limitations associated with the embryonic absence of Reelin), we used an inducible conditional mouse model (*UbiCRE*-*Reelin^F/F^*) in which Reelin is ubiquitously depleted, in a time-controlled manner, upon tamoxifen (TMX) administration.

## Results

### Postnatal Reelin inactivation leads to aberrant cortical and cerebellar lamination

To determine the role of Reelin in early postnatal stages, TMX was administered to *UbiCRE*-*Reelin^F/F^* postnatal day (P)1-3 mice (Fig. S1a) and pups were analyzed at several timepoints (P0-15). Western blot (WB) and immunohistochemical analyses indicated that Reelin levels were decreased by P3-4 and disappeared by P6, while Dab1 protein levels increased over time (Fig. S1b and c). NeuN immunohistochemistry at P15 revealed a marked disorganization of the upper neocortical layers through the entire rostrocaudal and mediolateral axes of cerebral cortex after postnatal inactivation of Reelin (Figs. 1a and S2d). Upper layer-specific staining (Cux1+) showed a striking invasion of neurons into layer I of the neocortex, as well as an upward displacement of layers II–IV neurons (Figs. 1b, c and S2e). Particular regions, such as the cingulate cortex, showed a greater cell dispersion in *UbiCRE*-*Reelin^F/F^* mice (Fig. 1d). Analyses of the inner (Tbr1+) and lower cortical (CTIP2+) layers revealed no significant differences between *Reelin^F/F^* and *UbiCRE*-*Reelin^F/F^* mice, suggesting that Reelin deficiency at early postnatal stages specifically disrupted the migration and positioning of late-born neurons (Fig. S2).

**Figure 1:**
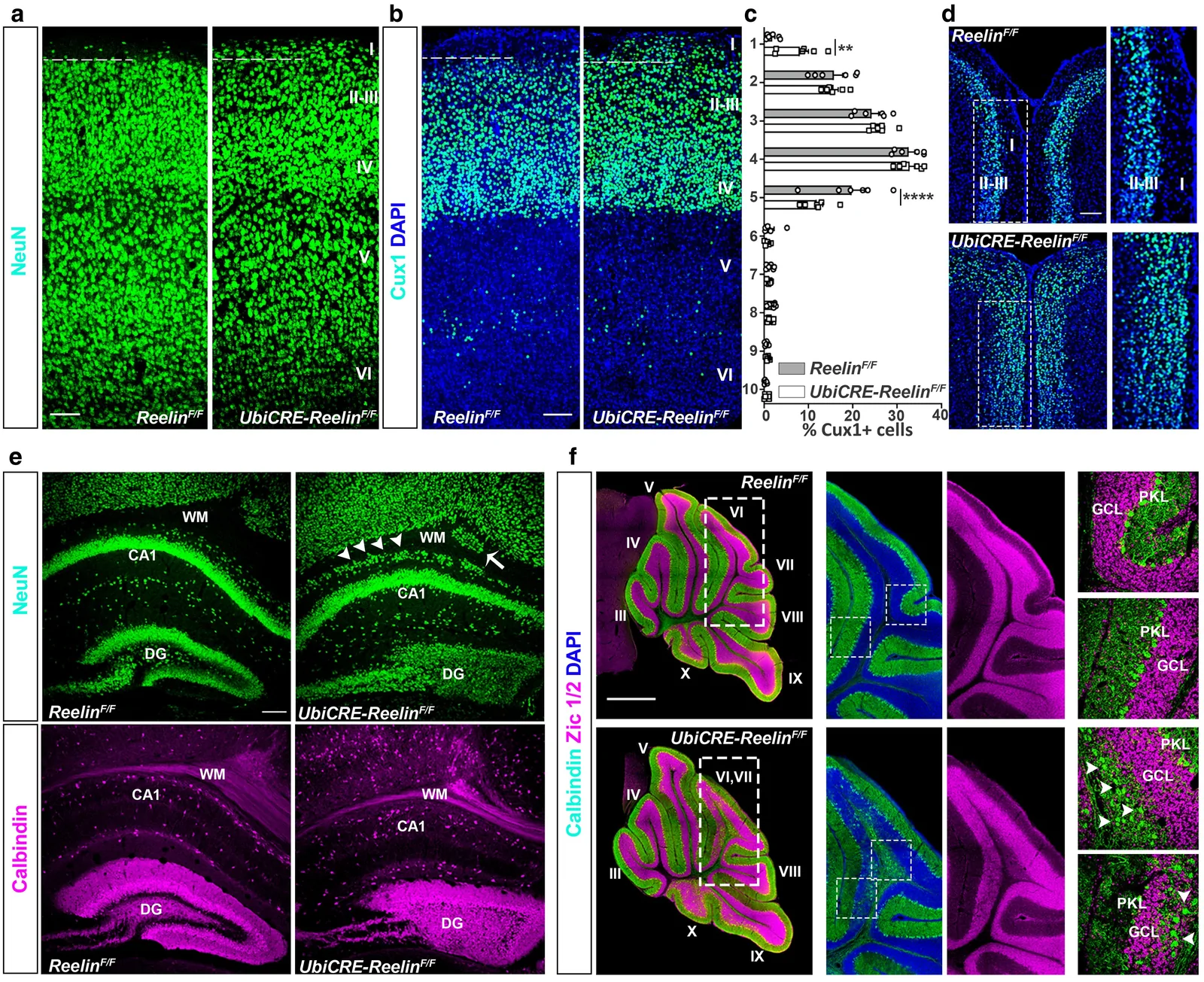
*Reln* inactivation at early postnatal stages (P1–3) critically affects brain cytoarchitecture. a, b, d) Representative confocal images depicting the distribution of NeuN+ (a) and Cux1+ (b, d) cells in the somatosensory (a, b) and cingulate (d) cortices of P15 *Reelin^F/F^* and *UbiCRE-Reelin^F/F^* mice. Note the striking upper-layer neuron invasion into layer I in the *UbiCRE-Reelin^F/F^* model. c) Percentage of Cux1+ cells per bin in the somatosensory cortex. e) Representative hippocampal NeuN and Calbindin staining images of *Reelin^F/F^* and *UbiCRE-Reelin^F/F^* P15 mice showing a failure to form the DG blades and a wider cell distribution in the absence of Reelin. Arrowheads and arrows indicate ectopic neurons in the stratum oriens and the WM, respectively. f) Representative Calbindin and Zic1/2 staining confocal images of the cerebellum vermis in *Reelin^F/F^* and *UbiCRE-Reelin^F/F^* P15 mice evidencing the defective positioning of PKc in the latter. Arrowheads indicate ectopic cells. Data represent mean ± SEM. Statistical analysis: two-way ANOVA with Bonferroni post hoc test. c) bin × genotype interaction *F*(9, 100) = 4.322 *P* < 0.0001; **, *P* < 0.01; ****, *P* < 0.0001; *n* = 6 mice per genotype. Scale bar, a, b, d, e) 100 µm, F, 700 µm. Cortical layers I to VI (I, II, III, IV, V, VI), cornus ammonis 1 (CA1), dentate gyrus (DG), white matter (WM), cerebellar lobes III–X (III–X), granular cell layer (GCL), and Purkinje cell layer (PKL).

The hippocampus also showed major alterations in *UbiCRE*-*Reelin^F/F^* mice. The dentate gyrus (DG) was dramatically disorganized, with granular cells failing to form the infra- and suprapyramidal blades and invading massively the hilar region. The cornus ammonis (CA1–CA3) regions displayed defective lamination, with a remarkable neuronal dispersion and ectopic clusters of neurons in the stratum oriens and white matter (Fig. 1e).

Control *Reelin^F/F^* cerebellar sections showed typical foliation, and Purkinje cells (PKc, Calbindin 28k+) arranged in a single layer with the dendrites occupying the molecular layer. The cerebella of *UbiCRE*-*Reelin^F/F^* mice exhibited an apparently normal foliation but presented many ectopic PKc; this defect was most severe in the most lateral VI-VII/IX-X folia, which develop later than the vermis (Figs. 1f and S3). The disposition of Zic1/2+ granule cells was mildly altered in the cerebella of *UbiCRE*-*Reelin^F/F^* mice, mostly as a consequence of the accumulation of ectopic PKc in the granular layer; there was no apparent loss of granule cells (Figs. 1f and S3). Together, these results indicate that early postnatal Reelin is essential for the correct migration of late-born cortical neurons and for the proper positioning of PKc, suggesting that Reelin is necessary for all phases of neuronal migration, including late migratory processes. These findings agree with a recent study demonstrating that inactivation of the *Reln* gene in GABAergic interneurons (mainly postnatally) results in cortical lamination deficits and invasion of layer I (4).

### Inactivation of the *Reln* gene at adult stages leads to disruption of cortical and cerebellar cytoarchitecture

We next investigated the effect of *Reln* inactivation during adulthood. Beyond P60, we observed a gradual decrease in Reelin levels, as measured by WB and immunohistochemistry, in *UbiCRE*-*Reelin^F/F^* mice not injected with TMX. This suggests a certain degree of leakage in these animals, precluding their use as controls. Consequently, *Reelin^F/F^* mice were used as controls and compared to TMX-injected *UbiCRE*-*Reelin^F/F^* littermates (Fig. S4; see Design of experimental groups, Materials and methods). P45–60 mice were injected with TMX and analyzed either 3–4 (Figs. 2, S5, and S6) or 11–12 (Figs. 2n, o and S7) months later. WB and immunohistochemistry confirmed that Reelin was depleted in brain samples from *UbiCRE*-*Reelin^F/F^* mice, concomitantly with an increase in Dab1 levels; no expression changes were detected in the two main Reelin receptors (VLDLR and ApoER2) (Fig. S5a–c). NeuN staining of adult *UbiCRE*-*Reelin^F/F^* neocortex revealed an interesting phenotype consisting of the dispersion of adult postmitotic neurons that distinctively invaded layer I (Figs. 2a, S6a, and S7e). This phenotype was observed at both ages studied, indicating that changes occur shortly after Reelin depletion and persist throughout the rest of adult life. The invasion of neurons in layer I was often uneven; intermittent clusters of neurons conferred an irregular appearance to the layer I/layer II interphase of *UbiCRE*-*Reelin^F/F^*, in contrast to the sharp interphase observed in control *Reelin^F/F^* mice. Although present throughout the neocortex, these ectopic aggregates in layer I were particularly prominent in the prefrontal, cingulate and somatosensory cortices (Figs. 2a, b, f, S6a, and S7e). Neurons ectopically positioned in layer I corresponded to upper layer neurons (Cux1+); an upward displacement of layers II–IV neurons was also detected. Analyses of early-born neurons (CTIP2+) also revealed a slight upward displacement in their cortical distribution which was statistically significant in the 5- to 6-month-old group (Figs. 2b–e, S6b, and S7b–d).

**Figure 2:**
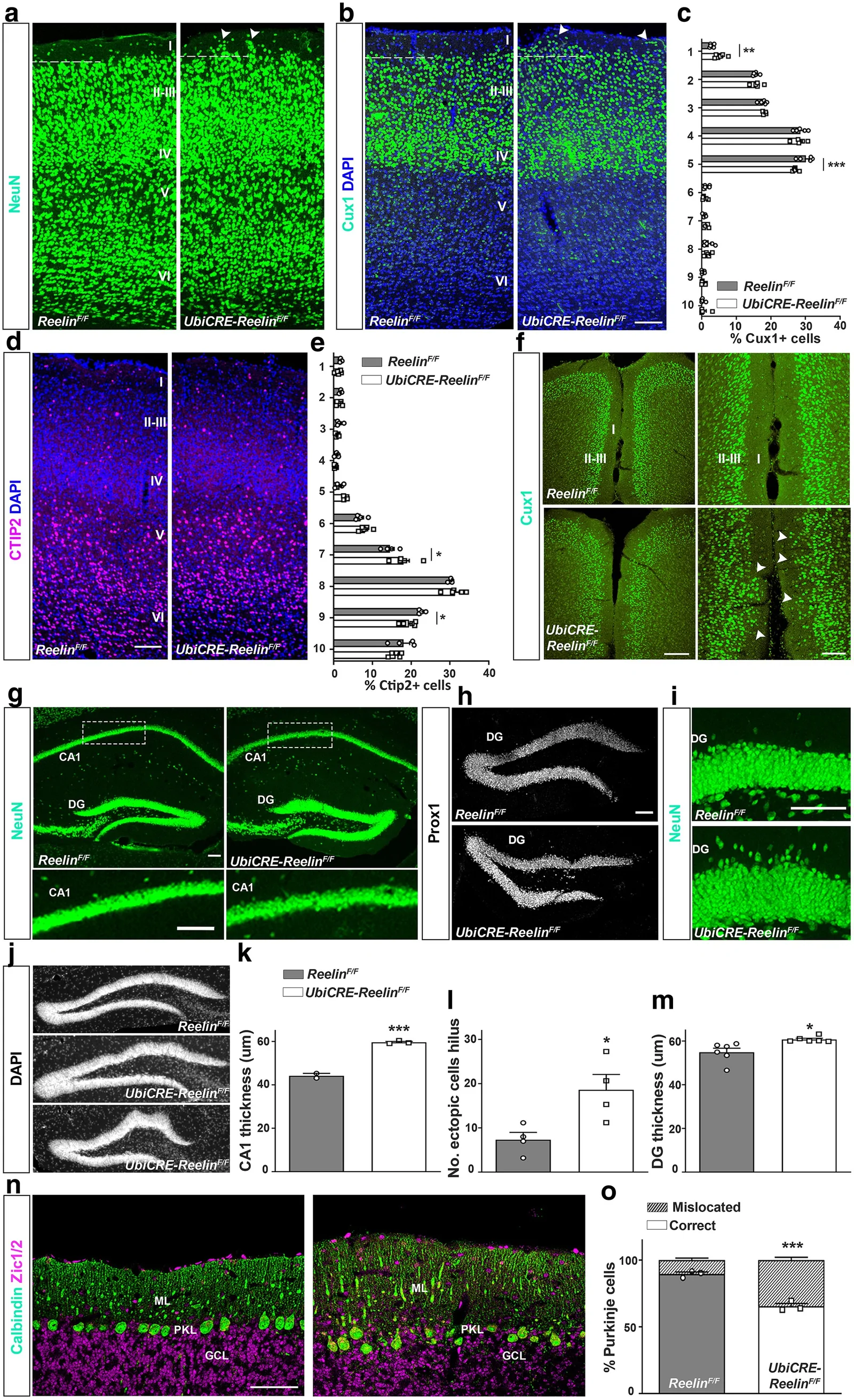
Inactivation of *Reln* in the adult brain results in neuronal dispersion. a, b, d, f) Representative confocal images illustrating the distribution of NeuN+ (a), Cux1+ (b, f) and CTIP2+ (d) cells in somatosensory (a, b, d) and cingulate (f) cortices of adult (5- to 6-month-old) ReelinF*^/F^* and *UbiCRE-Reelin^F/F^* mice. Note the presence of intermittent clusters of upper-layer neurons invading layer I (arrowheads), and the laminar alterations in the remaining layers. c, e) Percentage of Cux1+ (c) and CTIP2+ (e) cells per bin in the somatosensory cortex. g) NeuN immunostaining evidencing the wider pyramidal layer distribution in hippocampi of *UbiCRE-Reelin^F/F^* mice. h) DG Prox1+ immunostaining in *Reelin^F/F^* and *UbiCRE-Reelin^F/F^* mice evidencing granule cell dispersion and a remarkable presence of ectopic cells in the hilus of *UbiCRE-Reelin^F/F^* mice. i, j) Representative DG images showing a wider granule cell distribution and the presence of protruding areas in the *UbiCRE-Reelin^F/F^* model. k) Analysis of CA1 thickness. l) Quantification of the number of Prox1+ cells mislocated to the hilus. m) Analysis of DG thickness. n) Representative Calbindin and Zic1/2 confocal images of 12- to 13-month-old *Reelin^F/F^* and *UbiCRE-Reelin^F/F^* mouse cerebella showing PKc mispositioning in the latter model. o) Percentage of mislocated PKc. Data represent mean ± SEM. Statistical analyses: two-way ANOVA with Bonferroni post hoc test, c) bin × genotype interaction *P* = 0.0009 *F*(9, 80) = 3.569; *n* = 5 mice per genotype, e) bin × genotype interaction *P* = 0.0015, *F*(9, 70) = 3.420; *n* = 4–5 mice per genotype; unpaired two-tailed Student's t test, k) *t* = 16.01 df = 3 *P* = 0.0005, *n* = 2–3 mice per genotype, l) *t* = 3.452 df = 6 *P* = 0.0136, *n* = 4 mice per genotype, m) *t* = 2.929 df = 6 *P* = 0.0263, n = 6 mice per genotype, o) *t* = 9.579 df = 4 *P* = 0.0007, *n* = 3 mice per genotype. *, *P* < 0.05; **, *P* < 0.01; ***, *P* < 0.001. Scale bar, b, d, h, i) 100 µm, F, 200 µm and 100 µm for low- and high-magnification images, g) 100 µm and 50 µm for low- and high-magnification images, n) 700 µm. Cortical layers I to VI (I, II, III, IV, V, VI), cornus ammonis (CA), dentate gyrus (DG), granular cell layer (GCL), molecular layer (ML), and Purkinje cell layer (PKL).

To confirm the identity of the ectopic neurons invading layer I in the absence of Reelin, birth-dating experiments were performed using BrdU pulses at E15–16 to label upper layer neurons. The ectopic neurons invading layer I after *Reln* inactivation at either postnatal or young adult stages were co-labeled with Cux1 and BrdU, thus confirming that they were late-born cortical neurons (Fig. S8a and b). Finally, administration of multiple BrdU injections (P67–P74) after adult *Reln* inactivation at P60 ruled out the incorporation of adult-born neurons into layer I (Fig. S8c).

Analyses of the hippocampus of *UbiCRE*-*Reelin^F/F^* mice with NeuN staining indicated a thicker pyramidal layer in the CA1–CA3 regions, which often presented uneven edges compared with those of *Reelin^F/F^* controls (Figs. 2g, k and S7f, g). The dentate granule cell layer was also thicker and presented expansions and numerous ectopic granule cells in the molecular layer, further suggesting granule cell dispersion in the absence of Reelin (Figs. 2h–j, m and S7h, i). Analyses with the granule cell marker Prox1 showed an increased number of ectopic Prox1+ cells in the hilus of *UbiCRE*-*Reelin^F/F^* brains. Together, these data suggest a role for Reelin in granule cell distribution and maintenance of adult DG layers (Fig. 2h and l).

We next examined the cerebellum and found no signs of abnormal foliation after adult *Reln* inactivation. Nevertheless, a clear disruption of the PKc layer was observed in *UbiCRE*-*Reelin^F/F^* mice, with more than 30% of the PKc being mislocated (Fig. 2n and o). However, no motor function impairments were found in *UbiCRE*-*Reelin^F/F^* compared with *Reelin^F/F^* mice (Fig. S9a–e).

### Inactivation of the *Reln* gene at adult stages alters oscillatory network activity

To investigate the functional synaptic effects of adult *Reln* inactivation, input/output curves and LTP evoked at the hippocampal CA3–CA1 synapse were recorded in alert, behaving *Reelin^F/F^* and *UbiCRE*-*Reelin^F/F^* mice (7) (Fig. 3a). The synaptic activity of CA1 pyramidal neurons in response to single pulses of increasing intensity (0.02–0.2 mA) presented to CA3 Schaffer collaterals showed increases in the amplitude of evoked field excitatory postsynaptic potentials (fEPSPs) with increasing intensities. Although *Reelin^F/F^* mice reached larger fEPSP amplitudes faster than *UbiCRE*-*Reelin^F/F^* mice did, no significant differences were observed, indicating normal basic excitability of CA3–CA1 synapses in the absence of Reelin (Fig. 3b). We next evoked LTP at the CA3–CA1 synapse. After collection of baseline fEPSPs (3 times/min) during 15 min, animals were presented with a high-frequency stimulation (HFS) protocol, followed by a recording session where single stimuli were presented (3 times/min) and fEPSP collected during 60 min (Fig. 3c). Recording sessions (30 min each) were repeated for four days. Even though both groups exhibited a significant LTP following HFS, *UbiCRE*-*Reelin^F/F^* mice presented a significantly larger and longer lasting LTP than their littermate controls (Fig. 3c).

**Figure 3:**
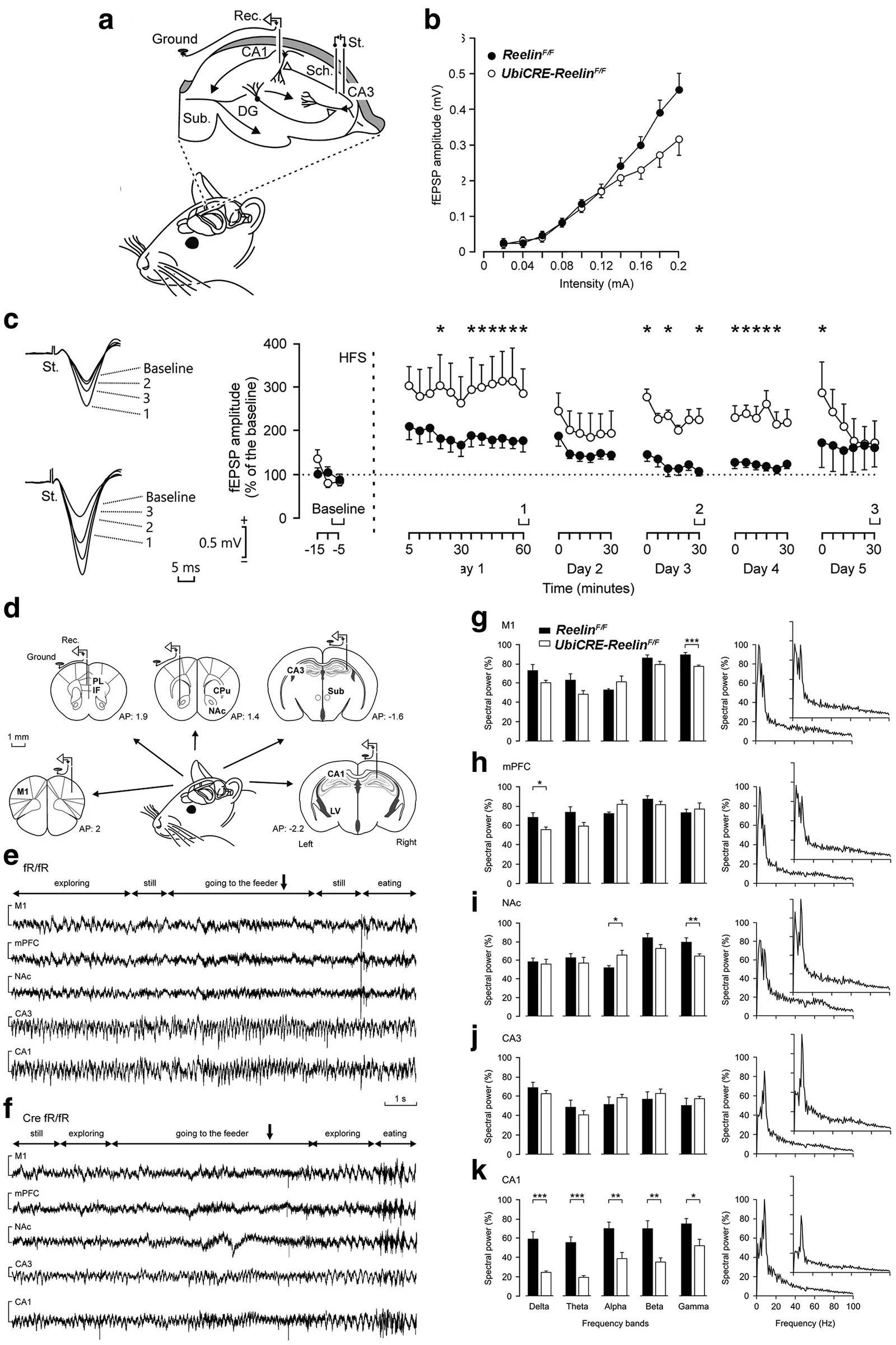
*Reln* inactivation during adult stages results in enhanced LTP and altered cortical rhythmicity. a) Animals were implanted with stimulating (St) electrodes on CA3 Schaffer collaterals and with a recording (Rec.) electrode in the ipsilateral CA1 area. b) Input/output curves of fEPSPs evoked at the CA3–CA1 synapse in alert behaving mice. Although fEPSP amplitudes increased with stimulus intensities, no significant differences were observed between genotypes. c) At the top are illustrated examples of fEPSP collected at the indicated times from an animal per genotype. LTP was evoked by a HFS session and followed for 5 days. Both genotypes showed significant LTPs when HFS-evoked fEPSPs were compared with baseline values. The *UbiCRE-Reelin^F/F^* group presented a significantly larger and longer lasting LTP. Although the groups × sessions analyses rendered no significant differences, a subsequent analysis indicated the presence of significant differences across sessions. d) Diagram of the location of the implanted electrodes for LFP recordings in *Reelin^F/F^* and *UbiCRE-Reelin^F/F^* mice. e, f) Representative examples of LFPs collected from *Reelin^F/F^* and *UbiCRE-Reelin^F/F^* mice during a behavioral sequence that included an approach to the feeder and the collection of the available pellet of food. From top to bottom: behavioral sequence and LFPs collected from the motor cortex (M1), medial prefrontal cortex (mPFC), nucleus accumbens (NAc), and hippocampal CA3 and CA1 areas. Eating is identified by the fast EMG activity (with a 6–7 Hz envelope) evoked at the masticatory muscles. Vertical arrows indicate when the animal crossed a light beam to approach the feeder. Calibration mark is 0.5 mV and 1 s for all traces. g–k) Spectral analyses of LFPs collected from *Reelin^F/F^* and *UbiCRE-Reelin^F/F^* mice during the performance of a behavioral sequence similar to that in (e, f). Spectral analyses were extracted from 15 s segments (*n* = 10 per group) of LFPs. The relative spectral powers of delta (0–5 Hz), theta (5–7 Hz), alpha (7–12 Hz), beta (12–30 Hz), and gamma (30–100 Hz) bands are illustrated for LFPs recorded in M1 (g), mPFC (h), NAc (i), CA3 (j), and CA1 (k). The complete spectral powers (1–100 Hz) corresponding to recordings collected from the five areas mentioned are illustrated at the right. Note that LFPs recorded from *UbiCRE-Reelin^F/F^* mice presented significant lower spectral power in the gamma-band of M1 and NAc, in the delta-band of mPFC, in the alpha-band of NAc and in all analyzed bands of CA1. Data represent mean ± SEM. Statistical analyses: repeated measures two-way ANOVA with Holm–Sidak multiple comparison b, *P* = 0.667 *F*(9, 99) = 0.745; *n* = 6–7 mice per genotype c, compared to baseline values *P* < 0.001 *F*(38, 228) = 2.719; *Reelin^F/F^* vs. *UbiCRE*-*Reelin^F/F^ P* = 0.006 *F*(1, 6) = 17.352; groups × session *P* = 0.886 *F*(38, 228) = 0.722; followed by multiple comparison with Holm–Sidak method; *t* = 4.166, *n* = 6–8 mice per genotype. Two-way ANOVA with Holm–Sidak multiple comparison, g–k) g) gamma-band of M1 *P* = 9.217 × 10^−4^  *F*(1,18) = 15.668; h) delta-band of mPFC *P* = 0.043 *F*(1,18) = 4.765; i) alpha-band of NAc [*P* = 0.035 *F*(1,18) = 5.227 and gamma-band of NAc *P* = 0.006 *F*(1,18) = 9.913; k) CA1 delta: *P* = 5.129 × 10–4 *F*(1,18) = 17.823; theta: *P* = 3.143 × 10–5, *F*(1,18) = 30.324; alpha: *P* = 0.006 *F*(1,18) = 9.727; beta: *P* = 0.002 *F*(1,18) = 12.705; gamma: *P* = 0.025 *F*(1,18) = 5.996. *n* = 7 mice per genotype. Primary motor cortex (M1), medial prefrontal cortex (Mpfc), nucleus accumbens (NAc), and cornus ammonis (CA).

Since Reelin deficits are associated with neuropsychiatric disorders (12, 13, 22), and alterations in cortical oscillatory rhythmicity are common in these diseases (23–25), we investigated to what extent rhythmic neural synchronization was altered in *UbiCRE*-*Reelin^F/F^* mice. We recorded local field potentials (LFPs) in behaving mice simultaneously in five different cortical (primary motor cortex [M1], medial prefrontal cortex [mPFC], CA3, and CA1), and subcortical (nucleus accumbens [NAc]) regions (Fig. 3d). LFP recordings were collected in 20-min sessions with animals located in an enriched arena, allowing exploration and feeding from a feeder dispensing food pellets randomly. Even though mice behaviors in the arena were similar, recorded LFPs presented interesting differences between genotypes. A quantitative analysis of the recorded power spectra indicated an overall larger spectral power in control *Reelin^F/F^* mice compared with that in *UbiCRE*-*Reelin^F/F^* mice (Fig. 3e and f). Significant differences were observed in the gamma band of M1 (Fig. 3g), in the delta band of mPFC (Fig. 3h), and in the alpha and gamma bands of NAc (Fig. 3i) recordings. Although no differences were observed in CA3 recordings (Fig. 3j), LFPs recorded in CA1 presented dramatic differences between the two genotypes in all analyzed bands (Fig. 3k). These data provide evidence that inactivation of the *Reln* gene at adult stages alters oscillatory network activity in different brain regions in vivo.

### Inactivation of the *Reln* gene during adulthood impairs long-term memory and alters depression-like behavior

Finally, we investigated whether the lamination defects associated with Reelin depletion during adulthood had any functional consequences. Passive avoidance testing showed no genotype differences 1 or 24 h after acquisition. However, Reelin deficiency significantly reduced the latency to enter the dark compartment 48 h after acquisition, suggesting impaired long-term memory consolidation in *UbiCRE*-*Reelin^F/F^* mice (Fig. 4a). Evaluation of depression-like behavior using the forced swim test (FST) showed that adult Reelin deficiency significantly increased floating time in *UbiCRE*-*Reelin^F/F^* mice, consistent with increased behavioral despair (Fig. 4b). However, no significant differences were observed in anxiety-like behavior, as measured using the black and white box (BWB) test (Fig. 4c) or in cocaine-induced psychostimulant sensitization, as assessed by locomotor exploration and sensitization responses (Fig. 4d).

**Figure 4:**
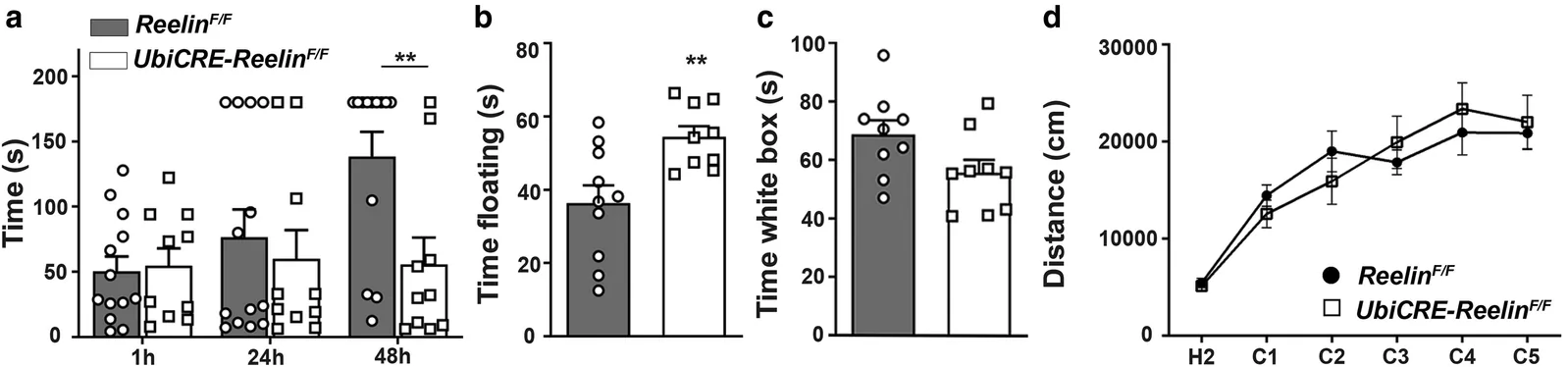
Adult *Reln* inactivation impairs memory and alters depression-like behavior. a) Latency to enter the dark compartment in the Passive Avoidance test. b) Time spent floating in the Forced Swim test. c) Time spent in the white box in the BWB test. d) Distance traveled in an open field after the administration of 10 mg/Kg of cocaine over 5 consecutive days. Data represents mean ± SEM and was analyzed with a two-way ANOVA with Bonferroni post hoc test (a, time × genotype interaction F (2,42) = 4.555 *P* = 0.0162 and d, n.s.) or an unpaired two-tailed Student's t test (b, *t* = 3.081 df = 17 *P* = 0.0068 and c, *t* = 1.986 df = 16 *P* = 0.0645); **P < 0.01. Seconds (s), habituation day 2 (H2), and cocaine day 1–5 (C1–C5).

## Discussion

This study shows that Reelin is required postnatally for the correct migration of cortical neurons and PKc in the cerebellum. Previous studies involving cell-autonomous inactivation of Reelin/Dab1 at perinatal stages (26) and, to a lesser extent, perinatal inactivation of GAD-derived Reelin (4) showed laminar deficits in the neocortex. To our knowledge this is the first demonstration that complete inactivation of the *Reln* gene at postnatal stages causes massive neocortical disruption. In addition, our findings in the hippocampus are entirely novel: perinatal Reelin inactivation in either GAD- or CR-cells leads only to mild lamination defects, likely due to compensation of the remaining Reelin expressing populations (4). Thus, the present study shows for the first time that total postnatal inactivation of the *Reln* gene leads to a *reeler*-like phenotype in the dentate gyrus and to substantial neuronal dispersion in the CA region.

Furthermore, we consider our findings in the cerebellum highly novel, as, to our knowledge, no previous study has described the consequences of postnatal Reelin inactivation in this region. Thus, postnatal inactivation of the *Reln* gene causes severe lamination alterations in different brain regions, with numerous mispositioned neurons.

Our study also highlights that adult Reelin is necessary to ensure the correct layering and cytoarchitectonic organization of the adult cerebral cortex and cerebellum. The wide extent of defects upon adult Reelin depletion, spanning all cortical regions examined and the cerebellum, raises the question as to whether control of adult cytoarchitecture by Reelin could similarly occur in nonlaminar brain nuclei, where Reelin also mediates developmental neuronal positioning (27).

Previous studies have reported occasional neuronal dispersion concomitant to Reelin deficits; particularly, granule cell dispersion in the dentate gyrus has been described in temporal lobe epilepsy, paralleled by a reduced Reelin expression (20, 21). However, to our knowledge, our study represents the first analysis reporting widespread dispersion of postmitotic neurons in adult brain, including neocortical neurons and PKc, after inactivation of a single gene. These results question the notion that the structural organization of the nervous system remains unchangeable once development has been completed. Thus, our data suggest that, in addition to structural and physiological plasticity of synapses, there may be substantial structural plasticity linked to neuronal dispersion and/or translocation. From a functional and mechanistic perspective, our findings raise interesting questions to be clarified, such as the identification of the molecular mechanisms involved, the likely concomitant circuitry changes, and their functional and physiological significance for adult brain function. Future studies will determine the molecular changes mediating such neuronal dispersion, including adhesion, migration mechanism or transcriptomic changes. In addition, our previous grafting experiments with Reelin+ cells, which prevented the invasion of layers II and III neurons into layer I, suggest that Reelin might act as a stopping or stabilizing signal in the adult brain (4).

Studies inactivating Reelin (4, 28, 29) or components of the Reelin pathway at prenatal ages (26, 30–32) report invasion of layer I by postmitotic neurons fated to layers II and III. In addition, invasion of Cux1+ neurons into layer I has been described after perinatal Dab1 inactivation in endothelial cells (33) and after perinatal inactivation of *Reln* specifically in cortical GABAergic interneurons (4). Our results add to these studies indicating that layers II and III neurons appear to be especially susceptible to undergo aberrant neuronal dispersion processes, even after *Reln* inactivation at adult stages. In addition, we also show wide neuronal dispersion in CA1–CA3 pyramidal neurons, dentate GCs, and PKc.

Another study where Reelin was inactivated at adult stages did not report cortical neuronal dispersion. This discrepancy may be related to the different Cre lines used (34) or to the different neuroanatomical approaches employed as this study was fundamentally addressed to analyze Alzheimer's disease anatomical hallmarks. Besides, the present data agrees with that study showing increased LTP responses after adult inactivation of the *Reln* gene. Previous studies that used *Reeler*-like mutants with migration deficits, acute Reelin treatments in vivo or in vitro, or Reelin-overexpressing transgenics, have concluded that Reelin also potentiates LTP responses (7, 35–37). The reasons why either Reelin depletion or supplementation results in increased LTP responses remain unknown although this may be related to several factors: methodological issues (our recordings were in awake, behaving mice while most studies use in vitro slices), age-dependent-inactivation specific effects, or most likely, compensatory molecular and synaptic circuit remodeling that possibly takes place during the dispersion of CA1 pyramidal neurons.

Our findings indicate that Reelin contributes to oscillatory activity and neural rhythms. Although the mechanisms linking Reelin to rhythmic activity are not known, we propose altered synaptic function as the most likely cause of rhythmic activity disturbances, since Reelin controls synaptic stability and function in the adult brain (10, 11, 35–37). Synchronized network activity is essential for many brain functions, including sleep/wake cycles, learning and memory, and cognition (38–40), and is frequently disrupted in psychiatric (41–44) and neurodegenerative diseases (45, 46). The *Reeler* and *Reeler*-like mutant models are not suitable for behavioral testing due to their severe motor deficits. Hence, there is a clear need to identify suitable models to evaluate the functional consequences of Reelin deficiency, particularly in the context of Reelin function in the adult brain. Our inducible conditional transgenic model showed no signs of motor deficits, as evidenced by the absence of locomotor abnormalities. This model therefore allowed us to assess behavioral outcomes of adult Reelin depletion without the major motor confounds of constitutive Reelin-deficient models. In this context, adult Reelin deficiency increased floating time in the FST, a readout commonly associated with depression-like behavior but also interpreted as altered stress-coping behavior in autism-related contexts (47), and reduced passive avoidance latency at 48 h, suggesting impaired long-term memory consolidation. The reported physiological and behavioral alterations may be a consequence of either Reelin deficiency or neuronal dispersion, or both.

Given the relationship established between psychiatric and neurodegenerative pathologies and Reelin deficits (12, 48–50), and more recently with Alzheimer's disease (14, 15), our results suggest that Reelin could be involved in the pathogenesis of these diseases via the disturbance of synchronous network activity as one plausible mechanism. Similarly, our results raise the question as to whether cortical lamination deficits observed in some neuropsychiatric disorders arise during embryonic development or may be occurring at later postnatal and adult stages as a consequence of Reelin deficits. Finally, it would be interesting to explore possible lamination alterations in the neocortex and entorhinal cortex in the context of Alzheimer's disease, given the massive loss of Reelin+ neurons in these regions (15, 51, 52).

## Materials and methods

### Animals

Floxed Reelin (*Reelin*^F/F^) mice (4), were bred to hemizygous *Ubiquitous CreER^T2^* mice (B6. Cg-Tg (UBC-cre/ESR1)1Ejb/J, stock #008085, The Jackson Laboratory) (53) to generate conditional transgenic mice with tamoxifen-inducible Cre activity (*UbiCRE*-*Reelin^F/F^*) (54). Upon tamoxifen administration, Cre-mediated recombination resulted in the deletion of the floxed sequence (*Reelin* exon1) from virtually all tissues (Fig. S1a). All mice used in the experiments were littermates and were kept on a C57BL/6J background and housed in groups in a 12 h light/dark cycle with free access to water and food. Unless otherwise stated, all procedures were performed with mice from both genders according to the guidelines of the European Community Directive 2010/63/EU and were approved by the local ethics committee for Animal Experimentation of the University of Barcelona (CEEA, Barcelona, Spain).

### Mouse genotyping

Genomic DNA was extracted from mouse tail tips and standard PCR amplification was performed. To determine the number of floxed Reelin alleles, the following primers were used: A 5′ CGA GGT GCT CAT TTC CCT GCA CAT TGC 3′ and B 5′ CAC CGA CCA AAG TGC TCC AAT CTG TCG 3′. In mice homozygous for floxed *Reelin* (*Reelin*^F/F^), a single band at 613 bp was amplified. Mice heterozygous for floxed Reelin (*Reelin*^F/WT^) had an additional band at 496 bp. To determine the presence of Ubi-Cre, the following primers were used: Transgene Fw 5′ GCG GTC TGG CAG TAA AAA CTA TC 3′, Transgene Rv 5′ GTG AAA CAG CAT TGC TGT CAC TT 3′, Common Fw 5′ CTA GGC CAC AGA ATT GAA AGA TCT 3′, and Common Rv 5′ GTA GGT GGA AAT TCT AGC ATC ATC C 3′. Mice heterozygous for Cre (*UbiCRE*-*Reelin^F/F^*) had two bands amplified (324 and 100 bp) while mice without Cre only had the 324 bp band amplified.

### Design of experimental groups

To characterize the *UbiCRE*-*Reelin^F/F^* model, we performed Reelin ICC and WB at different ages. As expected, Reelin levels were comparable between *Reelin^F/F^* and *UbiCRE*-*Reelin^F/F^.* However, we noticed that beyond P60, Reelin levels progressively decreased in the nontreated *UbiCRE*-*Reelin^F/F^* mice compared to *Reelin^F/F^* (Fig. S4). The occurrence of Cre-mediated recombination in the absence of tamoxifen (leaking) has been previously described and is common in Cre-ERT2 lines (55–57) as well as in other Cre lines with ubiquitous expression (58) that have been used to generate inducible cre-mediated Reelin KO mice (34). Since our data seemed to suggest a certain degree of leaking in nontreated *UbiCRE*-*Reelin^F/F^* mice, we decided to not use this group as control to avoid confounding factors in the interpretation of our results. Moreover, to guarantee that the effects reported are due to the specific depletion of Reelin at adult stages, we always performed the TMX injections between p45 and p60, an age where our data indicate that the levels of Reelin and its downstream effector Dab1 are not significantly different between *Reelin^F/F^* and nontreated *UbiCRE*-*Reelin^F/F^* and the layer I cell density is similar between groups (Fig. S4).

### Tamoxifen administration

#### Early postnatal administration

To conditionally delete *Reelin* at early postnatal stages, mouse pups received a daily intragastric injection of tamoxifen (TMX; 1 mg/mL dissolved in 2.5% EtOH/97.5% peanut oil) at 0.05 mg/pup/day for 3 consecutive days from postnatal day (P)1 to P3. Mice were then sacrificed at different postnatal days ranging from P0 to P15.

#### Adult administration

To obtain temporally controlled ubiquitous Cre-mediated recombination in young adult (P20–30) and adult (>P45) *UbiCRE*-*Reelin^F/F^* mice, intraperitoneal TMX injections were administered for 3 consecutive days (30 mg/mL dissolved in 10% EtOH/90% sunflower oil) at 90 mg/kg/day or 180 mg/kg/day, respectively. Unless otherwise stated, mice were then sacrificed either at 3–4 months or at 11–12 months after TMX injection.

### Antibodies and reagents

The following commercial primary antibodies were used for immunohistochemistry: anti-Reelin (1:500 dilution, clone G10, MAB5364, Millipore), anti-Cux1 (upper layers II–IV; 1:250, SC-13024, Santa Cruz Biotechnology), anti-Tbr1 (inner cortical layers II, III, VI, 1:500, Millipore), anti-CTIP2 (lower layers V, VI, 1:500, ab18465, Abcam), anti-BrdU (1:250, OBT0030, Axyll, and Clone BU1/75 ab6326, Abcam), anti-Calbindin (1:2,500, 300, Swant), anti-Zic1/2 (1:500, kind gift of Dr. J. Aruga, RIKEN Brain Science Institute), anti-NeuN (mature neurons, 1:500, MAB377B, Millipore), and anti-Prox1 (1:10,000, kind gift of Dr. S. Pleasure, University of California, San Francisco). For western blot, anti-β-III-Tubulin (1:100,000, MMS-435P, Covance), anti-Reelin (1:4,000, clone G10, MAB5364, Millipore), anti-VLDLR (1:2,500, clone #2897, kind gift of Dr. J. Herz, University of Texas Southwestern Medical Center, Dallas), anti-ApoER2 (1:2,500, clone #2561, kind gift of Dr. J. Herz, University of Texas Southwestern Medical Center, Dallas), anti-Dab1 (1:2,000, U100P, ExAlpha), and anti-Actin (1:10,000, MAB1501, Millipore) were used.

Alexa Fluor fluorescent secondary antibodies were from Invitrogen. Biotinylated secondary antibodies were from Vector Labs. Streptavidin-biotinylated/horseradish peroxidase (HRP) complex for enhanced chemiluminescence (ECL) were from GE Healthcare. *F*(ab′)_2_ fragment antimouse IgG was from Jackson ImmunoResearch. The HRP-labeled secondary antibodies used for Western blot were from DAKO. Antibodies were used according to manufacturers’ recommendations.

TMX, peanut oil, sunflower oil, bromodeoxyuridine (BrdU), Hoechst 33342, diaminobenzidine reagent (DAB), and Eukitt mounting media were from Sigma-Aldrich. Mowiol was from Calbiochem. BCA Protein Assay was from Thermo Fisher.

### Tissue processing and immunohistochemistry

Mice were deeply anesthetized and transcardially perfused with 4% paraformaldehyde (PFA) in 0.1 M PBS. After perfusion, the brain was removed from the skull and postfixed in 4% PFA for 24 h at 4 °C. Brains were then cryoprotected with 30% sucrose in 0.1 M PBS, frozen and coronally sectioned on a freezing microtome (Leica SM2010R) at 30 μm thickness.

For immunodetection of antigens, brain sections were blocked for 2 h at room temperature (RT) with 10% normal goat or horse serum (NGS or NHS), *F*(ab′)_2_ fragment antimouse unconjugated IgG (1:300) (the latter when needed) in 0.2% gelatin 0.1 M PBS. For BrdU and CTIP2 detection, heat-mediated antigen retrieval was performed before the blocking step. Primary antibodies were incubated overnight at 4 °C. For an immunofluorescent readout, sections were incubated for 2 h at RT with Alexa Fluor secondary antibodies (1:500) in 5% NGS or NHS in 0.1 M PBS, then counterstained with Hoechst 33342 or DAPI for nuclei detection, mounted in Mowiol and stored at −20 °C. For a chromogenic readout, sequential incubations were performed with biotinylated secondary antibodies (1:200; 2 h at RT) and streptavidin-HRP (1:400; 2 h at RT) in 5% NGS or NHS in 0.1 M PBS. The staining was developed using a solution of DAB and H_2_O_2_. Finally, sections were dehydrated and mounted with Eukitt.

### Western blot

Mice were sacrificed by decapitation and the brains were quickly removed from the skull. After the cerebellum was discarded, the brain was split into hemispheres, frozen in liquid nitrogen and stored at −80 °C until use. Brain tissue was processed as previously described (7). Briefly, brains were homogenized in lysis buffer using a Polytron (Ika T10 Basic), sonicated and centrifuged to remove insoluble debris. Supernatant was collected and stored at −20 °C until use. A small fraction of the supernatant was used to determine protein concentration using the BCA protein assay.

Samples were resolved by SDS-polyacrylamide gels and transferred onto nitrocellulose membranes. Membranes were then blocked for 2 h at RT in TBST containing 5% nonfat milk, then incubated overnight at 4 °C with most of the primary antibodies except for anti-Tubulin and anti-Actin, which were incubated for 30 min at RT. After incubation with secondary HRP-labeled antibodies (1:2,000) for 1 h at RT, membranes were developed with the ECL system.

### BrdU birthdating experiments

To determine the origin of the mispositioned cells present in the layer I of *UbiCRE*-*Reelin^F/F^* mice, different experiments administering BrdU were performed. Timed matings were set up, with the morning after the mating day considered as embryonic day (E)0. Pregnant females from timed matings were given 3 consecutive daily pulses of BrdU (50 mg/Kg) between E15 and E16 to label the upper cortical layer cells. The resulting offspring were either injected with TMX at P1–3 (intragastric route; 0.05 mg/pup/day) and sacrificed at P15, or injected with TMX at P20–30 (intraperitoneal route; 90 mg/kg/day) and sacrificed at P50–60.

In addition, to prove that the mispositioned cells present in layer I were not newly born after TMX-induced Reelin inactivation, mice were injected with TMX for 3 consecutive days as of P60, then injected with BrdU twice a day (6 h interval) on days 4, 8, and 12 after TMX administration and sacrificed around P120.

### Image acquisition and data analyses

Bright field images were acquired with an Olympus D72 camera attached to a Nikon Eclipse E600 microscope. Fluorescent images were taken using a Leica SP2 confocal microscope and a Nikon Eclipse E600 microscope. Whole slice mosaic images were acquired either with an Olympus ScanR microscope or a Nanozoomer 2.0-HT (Hamamatsu Photonics). Image processing was done with FIJI (Image J).

To determine the layer distribution of cells in the neocortex (Cux1 labeling specific for upper layers, and CTIP2 for lower layers), fluorescent images from the primary somatosensory barrel field (SBF1) were taken using a Leica TCS SP2 confocal laser scanning microscope and 10×, 20×, or 40× oil-immersion objectives. For each mouse, a minimum of three confocal images were acquired and the maximum intensity z-projection of each image was used to define 3 regions of interest (ROIs) per image (9 ROIs per mouse in total). A ROI was defined as the area comprised in a 70 µm width column (medio-lateral axis), and a height equal to the distance from the corpus callosum to the upper edge of the marginal zone of the neocortex (ventricular-pial axis). Those ROIs were then divided in 10 identical bins using a macro created in Image J, and the number of cells in each bin was manually quantified. The mean number of cells per bin was calculated for each animal and expressed as a percentage of the total number of cells per ROI. In all the analyses, the number of total cells per strip and the cortical thickness were also assessed. In all cases, we found no differences between genotypes in any of the parameters analyzed: Cux1 P15 total cell number 191 ± 16 vs. 228 ± 28; *P* = 0.2814 *t* = 1.139 df = 10, cortical width 1156 ± 38 vs. 1247 ± 34 µm; *P* = 0.1022 *t* = 1.799 df = 10; Cux1 adult total cell number 125.7 ± 5.7 vs. 133.7 ± 4.4; *P* = 0.3015 *t* = 1.105 df = 8 and cortical width 1113 ± 25.1 vs. 1136 ± 23.3 µm; *P* = 0.5189 *t* = 0.6746 df = 8; Cux1 old adult total cell number 126 ± 22.37 vs. 115 ± 13.22; *P* = 0.2786 *t* = 0.9854 df = 9 and cortical width 1018 ± 75.33 vs. 1050 ± 54.18 µm; *P* = 0.4380 *t* = 0.8115 df = 9; *n* = 5–6 mice per genotype.

To analyze cell dispersion in the hippocampus, images were taken using a Leica TCS SP2 confocal laser scanning microscope and the thicknesses of the CA1 and DG were measured. For the CA1 analysis, a grid (15,000 µm^2^) was used to divide the image in squares, and the CA1 thickness was measured in the center of each square (7 to 12 width measurements were taken per CA1). A minimum of 2 hippocampi per animal were analyzed. For the DG thickness analysis, the length of the DG was divided into 100-μm segments, and granule cell layer thickness measurements were taken every 100 μm. Between 5 and 12 DGs were analyzed per animal. To analyze the DG granule cell layer in detail, Prox 1 (granule cell layer marker) immunostaining was used. For each mouse, 5–7 DGs were analyzed using the maximum intensity z-projections of the acquired confocal images. The number of Prox 1+ cells ectopically located in the hilus were quantified.

To determine the layer distribution of Purkinje cells in the cerebellum of old adult mice, images were taken using a Leica TCS SP2 confocal laser scanning microscope. Three-four cerebella images for mouse were analyzed using the maximum intensity z-projection of the acquired confocal images. For the analysis, first the Zic1/2 staining (granular cells) was used to delineate the limit between the granular and molecular layers. Next, the number of Purkinje cell somas (Calbindin+) was counted while distinguishing between the ones properly positioned, which formed a 20-µm thickness monolayer between the granular and the molecular layers, and the mislocated ones, which were clearly outside that monolayer. The proportion of properly positioned and mislocated cells was calculated as a percentage of the total number of cells counted per cerebellum.

### Behavioral analyses

Mice used for all behavioral analyses were adult males between 4 and 7 months of age.

#### Passive avoidance

The passive avoidance test was conducted in a cage with two compartments (light and dark) connected by a door. Initially, the mice were placed in the light compartment and allowed to freely explore for a maximum of 3 min. When the mice entered the dark compartment, they received an electric foot shock and the test ended. Mice were retested after 1, 24, and 48 h and the latency to enter in the dark compartment was measured (*n* = 13 *Reelin^F/F^* and *n* = 9 *UbiCRE-Reelin^F/F^* mice).

#### Forced swim test

The FST is used as a measure of depression-like behavior. In the FST, mice were placed for 5 min in a 5 L beaker (diameter: 20 cm; water depth: 12 cm) filled with warm water (27 °C) up to 3 L. The time that the animals were active was measured for the last 4 min of test and used to calculate the immobility time (Immobility time = 240-activity time; *n* = 10 *Reelin^F/F^* and *n* = 9 *UbiCRE-Reelin^F/F^* mice).

#### BWB test

In the BWB test, mice were placed in the dark compartment of a box (40 lux; 20 × 20 cm) connected to a well-lit, white compartment (400 lux; 25 × 25 cm) and allowed to freely explore the box for 5 min. The time the mice spent in the white compartment was measured (*n* = 9 *Reelin^F/F^* and *n* = 9 *UbiCRE-Reelin^F/F^* mice).

#### Cocaine sensitization

For the cocaine sensitization test mice were habituated to the open field box for 2 days. Starting the following day, mice were administered intraperitoneally with either cocaine (10 mg/kg in 0.9% NaCl) or saline (0.9% NaCl) and immediately placed in the OF apparatus. This procedure was repeated for 5 consecutive days, and the locomotion was recorded for 20 min using the Smart Junior software from Panlab (*n* = 10 *Reelin^F/F^* and *n* = 9 *UbiCRE-Reelin^F/F^* mice).

#### Open field test

An open field test was performed to determine the spontaneous motor activities of experimental animals (59). For this, we used an Actifot 809 (Cebertec, S.A., Madrid, Spain) open field arena measuring 28 × 28 × 21 cm and provided with infrared lights (every 2 cm) in the axes X, Y, and Z. The apparatus was located inside a dimly illuminated sound-proof room. Each mouse (*n* = 13 *Reelin^F/F^* and *n* = 9 *UbiCRE-Reelin^F/F^* mice) was gently place in the center of the box and its motor activity was quantified for 15 min. A computer program (MUX_XYZ16L from Cebertec) automatically quantified the distance (in cm) traveled by the animal during the test period. After each trial, the apparatus was cleaned with 70° proof alcohol.

#### Rotarod test

The rotarod test was carried out to determine the motor coordination of the two groups of animals (*n* = 13 *Reelin^F/F^* and *n* = 9 *UbiCRE-Reelin^F/F^* mice) (60). We used an accelerating rotarod treadmill (Ugo Basile, Varese, Italy). Each mouse was placed the first day (session 1) on the rod and tested at increasing speeds (2–20 rpm) for a maximum of 300 s. This same test was repeated twice with 1 h of interval. Animals were retested 24 h (session 2) and 72 h (session 3) later. The total time that each animal remained on the rod was computed as latency to fall, recorded automatically by a trip switch under the floor of each rotating drum. Results were evaluated by averaging the data collected from each of the three sessions.

#### Elevated wire and path tests

These two tests were designed to further check motor abilities of the animals in difficult tasks. The elevated wire consisted of a 40 cm long, 1 mm wide stainless-steel wire located 60 cm over a soft cushion, while the elevated bar substituted the wire for 1 cm wide plastic bar. Each mouse was placed in the center of the elevated wire or bar and allowed a maximum of 30 s to reach one of the platforms (12 × 12 cm) located at each end. The latency to fall was quantified (test 1). The test was repeated 24 h later (test 2). A total of *n* = 13 *Reelin^F/F^* and *n* = 9 *UbiCRE-Reelin^F/F^* mice were used in these two tests.

### Animal preparation for electrophysiological experiments

Electrophysiological experiments were carried out in the animal facilities of the Division of Neurosciences (Pablo de Olavide University, Seville, Spain). Male *Reelin^F/F^* and *UbiCRE*-*Reelin^F/F^* mice were provided by the Barcelona Science Park Animal House (Barcelona) and were 3–5 months old upon their arrival to the Pablo de Olavide University Animal House. Animals were maintained in collective cages, but after surgery, they were kept in individual cages until the end of the experiments. Mice were maintained on a 12-h light/dark cycle with constant temperature (21 ± 1 °C) and humidity (50 ± 5%) and they were allowed access ad libitum to commercial mouse chow and water. Experiments were performed following European Union (2010/63/EU) and Spanish (BOE 34/11370-421, 2013) regulations for the use of laboratory animals in chronic experiments. In addition, all experimental protocols were approved by the Ethics Committee of the Pablo de Olavide University (JA/CAPD 06/03/2018/025).

For electrode implantation, animals were anesthetized with 0.8–3% halothane delivered from a calibrated Fluotec 5 (Fluotec-Ohmeda, Tewksbury, MA, USA) device via a home-made mask and vaporizer set up at a flow rate of 0.8 L/min oxygen. Animals were implanted with single recording electrodes at the right M1 (AP: 2; L: 1.7; and D: −1), left mPFC (AP: 1.9, L: −0.3, D: −1.6), left NAc (AP: 1.4, L: −0.9, D: −4.0), right CA1 area (AP: −2.2; L: 1.5; D: −1.3) (61). Finally, the right hippocampal CA3 area (AP: −1.5; L: 1.7; D: −1.5) was implanted with a pair of recording and stimulating electrodes. Electrodes were made of 50-μm Teflon-coated tungsten wire (Advent Research Materials Ltd., Eynsham, England). Two bare silver wires (0.1 mm) were affixed to the skull as ground. Wires were soldered to two 4-pin sockets. Sockets were fixed to the skull with the help of three small screws and dental cement (7, 62).

### LFP recordings carried out in an enriched arena

We recorded LFPs evoked in different cortical (mPFC, M1, CA3, and CA1) and subcortical (NAc) structures during the spontaneous activity of the experimental animals located in an enriched arena. The arena consisted of a box (30 × 20 × 15 cm) provided with nesting material and different objects, and it included a corridor ending at a feeder; the corridor was provided with a light beam located 10 cm from the feeder (63). To avoid noisy interferences, the arena was housed within a sound-attenuating chamber (90 × 55 × 60 cm), dimly illuminated (19 W lamp) and exposed to a 45 dB white noise (Cibertec, S.A., Madrid, Spain). Each animal was placed in the arena 20 min/day for up to 10 recording sessions. LFPs were recorded with Grass P511 differential amplifiers across high impedance probes (2 × 1012 Ω; 10 pF), and with a bandwidth of 0.1 Hz–10 kHz (Grass-Telefactor, West Warwick, RI, USA). Sessions were recorded with a video-capture system (Sony HDR-SR12E, Madrid, Spain) synchronized to LFP recordings.

### Input/output curve and LTP experiments in behaving mice

For these electrophysiological studies, mice were located in individual small (5 × 5 × 5 cm) boxes, aimed to avoid excessive walking (7, 62). Boxes were located in a larger (40 × 30 × 20 cm) Faraday box.

For input/output curve experiments, mice (*n* = 7 *Reelin^F/F^* and *n* = 6 *UbiCRE*-*Reelin^F/F^* mice) were stimulated at the Schaffer collateral/commissural pathway nearby CA3 somas with 10 single pulses (100 μs, square, biphasic pulses) at increasing intensities (0.02–0.2 mA), while recordings were carried out at the ipsilateral stratum radiatum underneath the CA1 area.

For the LTP experiments, we used *n* = 8 *Reelin^F/F^* and *n* = 6 *UbiCRE*-*Reelin^F/F^* mice. Baseline fEPSP values evoked at the CA3–CA1 synapse were collected 15 min prior to LTP induction using the same single pulses presented every 20 s. Pulse intensity was set at 30–40% of the intensity necessary for evoking a maximum fEPSP response (0.15–0.25 mA) (62, 64). LTP was evoked with a high-frequency stimulus (HFS) protocol consisting of five 200 Hz, 100-ms trains of pulses presented at a rate of 1/s, and repeated six times, at intervals of 1 min. The stimulus intensity during the HFS protocol was set at the same value as that used for generating baseline recordings; in this way, we prevented the presentation of unwanted seizures and/or large population spikes. After the HFS session, the same single pulses were presented every 20 s for 0 additional min on that same day, and for 30 min on the following 4 days (7, 62). For both input/output curves and LTP were recorded as described above.

### Histology after electrophysiological experiments

At the end of the electrophysiological experiments, animals were deeply reanesthetized (sodium pentobarbital, 50 mg/kg) and perfused transcardially with saline and 4% phosphate-buffered paraformaldehyde. Their brains were removed, postfixed overnight at 4 °C, and cryoprotected in 30% sucrose in PBS. Sections that included the implanted sites were obtained in a microtome (Leica, Wetzlar, Germany) at 50 μm thickness, mounted on gelatinized glass slides, and stained with 0.1% toluidine blue to determine the proper location of stimulating and recording electrodes (7, 62).

### Electrophysiological experiment data collection and analyses

LFPs, fEPSP recordings, and 1V rectangular pulses corresponding to stimulus presentations and light-beam crossings were stored digitally on a computer through an analog-to-digital converter (CED 1401 Plus; Cambridge Electronics Design, Cambridge, UK). Data were analyzed offline for quantification of LFPs and fEPSPs with the Spike 2 software (Cambridge Electronics Design) and the video-capture system.

For LFP analysis, we used 15-s epochs collected from sessions presenting good quality records and different behaviors, which usually included crossing the light beam to reach the feeder. Analytical procedures were developed in a previous study from some of us with the help of MATLAB (version 9.4, R2018a, The MathWorks, Natick, MA, USA) routines and customized scripts of Chronux software (versions 2.11/R2014 and 2.12/R2018. Website: http://chronux.org/) (65). We selected the following frequency bands: delta, 0–5 Hz; theta, 5–7 Hz; alpha, 7–12 Hz; beta, 12–30 Hz; and gamma, 30–100 Hz. The algorithm included the analysis of mean values of the spectral powers between the different frequency bands inside each epoch, and the analysis of mean values of the spectral powers for the same frequency band between the different epochs (65). For input/output curves and LTP analyses, five successive fEPSPs were averaged, and the mean value of the amplitude (in mV) was determined.

### Statistical analyses

Sample sizes are detailed in each figure legend and data are presented as mean ± SEM. Quantification of immunostaining was performed blindly. All statistical analyses were performed using either GraphPad Prism 6.0 software or the Sigma Plot 11.0 package (Sigma Plot, San Jose, CA, USA) and the Statistics MATLAB Toolbox (LFP spectral powers and the fEPSP synapsis strengths). Data were tested using two-tailed unpaired t-test and one- or two-way ANOVA with or without repeated measures followed by the corresponding pairwise multiple comparison procedures. When the normality test failed, an ANOVA test on ranks (i.e. the nonparametric version of the classical ANOVA *F*-test) was applied (particular tests for each experiment are mentioned in figure legends). Statistical significance was established at *P* < 0.05.

## Supplementary Material

pgag363_Supplementary_Data

## Data Availability

All study data are included in the article and/or SI Appendix.
