# Supplementary material for: Adult inactivation of the Reln gene leads to disruption of cortical lamination and rhythmic activity
Source: PNAS Nexus. 2026 Oct 29;5(11):pgag363. doi: 10.1093/pnasnexus/pgag363 (PMC13741246; doi:10.1093/pnasnexus/pgag363)
Supplement: Supplementary file 1 [file pgag363_supplementary_data.pdf]

Supporting Information for

Adult inactivation of the *Reln* gene leads to disruption of cortical lamination and rhythmic activity

Yasmina Manso[1,2,#,*], Nuria Masachs[1,3,#], Alba Vílchez-Acosta[1,2,#], Victoria González[1], Gerardo Contreras-Murillo[4], Raudel Sánchez-Campusano[4], Marc Hernáiz[1], Alba Elias-Tersa[1,2], María Ángeles Llanos[1], Gerard Rovira-Escalé[1], Ana Guerrero[1,2], Magdalena Martínez-Losa[5], Manuel Álvarez-Dolado[5] , José María Delgado-García[4], Angus C. Nairn[6], Agnès Gruart[4], Eduardo Soriano[1,2,*]

[1] Department of Cell Biology, Physiology and Immunology, Faculty of Biology and Institute of Neurosciences, University of Barcelona, 08028 Barcelona, Spain

[2] Centro de Investigación Biomédica en Red Enfermedades Neurodegenerativas (CIBERNED), ISCIII, 28031 Madrid, Spain

[3] Current address: INSERM, Magendie, U1215, University of Bordeaux, F-3300 Bordeaux, France.

[4] Division of Neurosciences, Pablo de Olavide University, 41013 Sevilla, Spain

[5] Andalusian Center for Molecular Biology and Regenerative Medicine-CABIMER, CSIC, Seville 41092, Spain.

[6] Department of Psychiatry, Yale University School of Medicine, New Haven, 06508 Connecticut

[#] These authors contributed equally to this work

[*] Corresponding authors: esoriano@ub.edu, ymansosanz@ub.edu

This PDF includes:

Figures S1 to S9

SUPPLEMENTARY FIG. 1

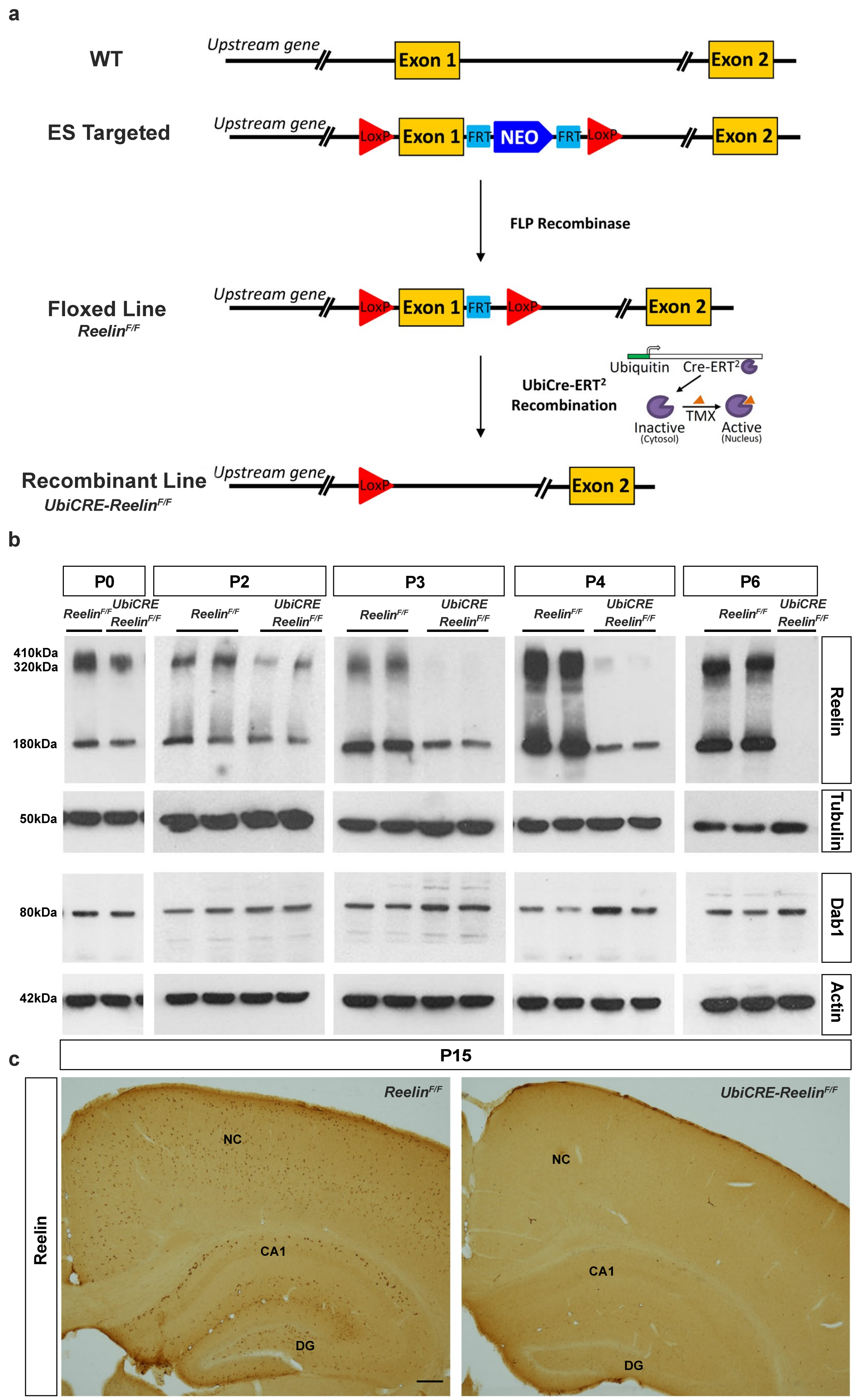

SUPPLEMENTARY FIG. 2

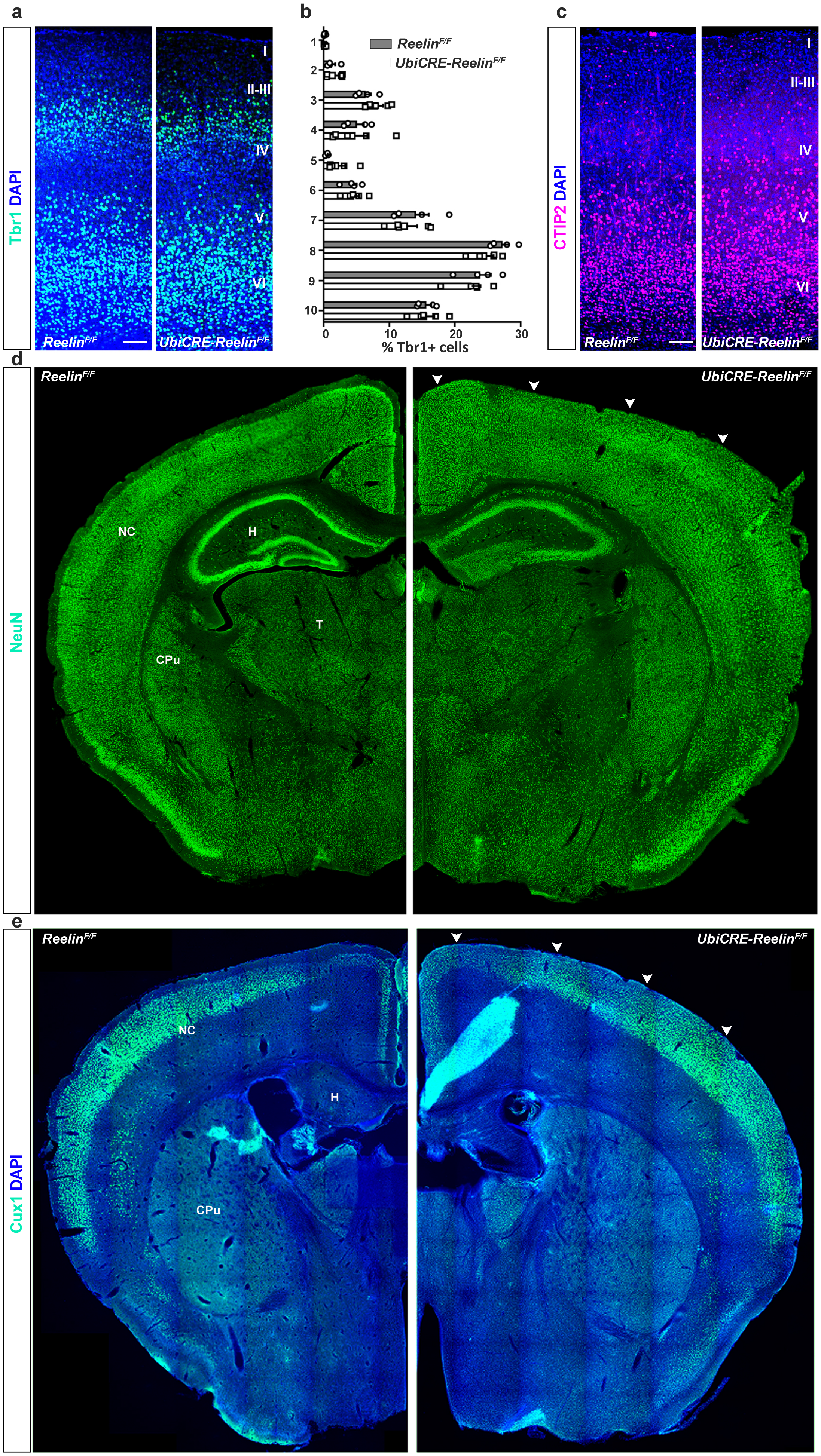

SUPPLEMENTARY FIG.3

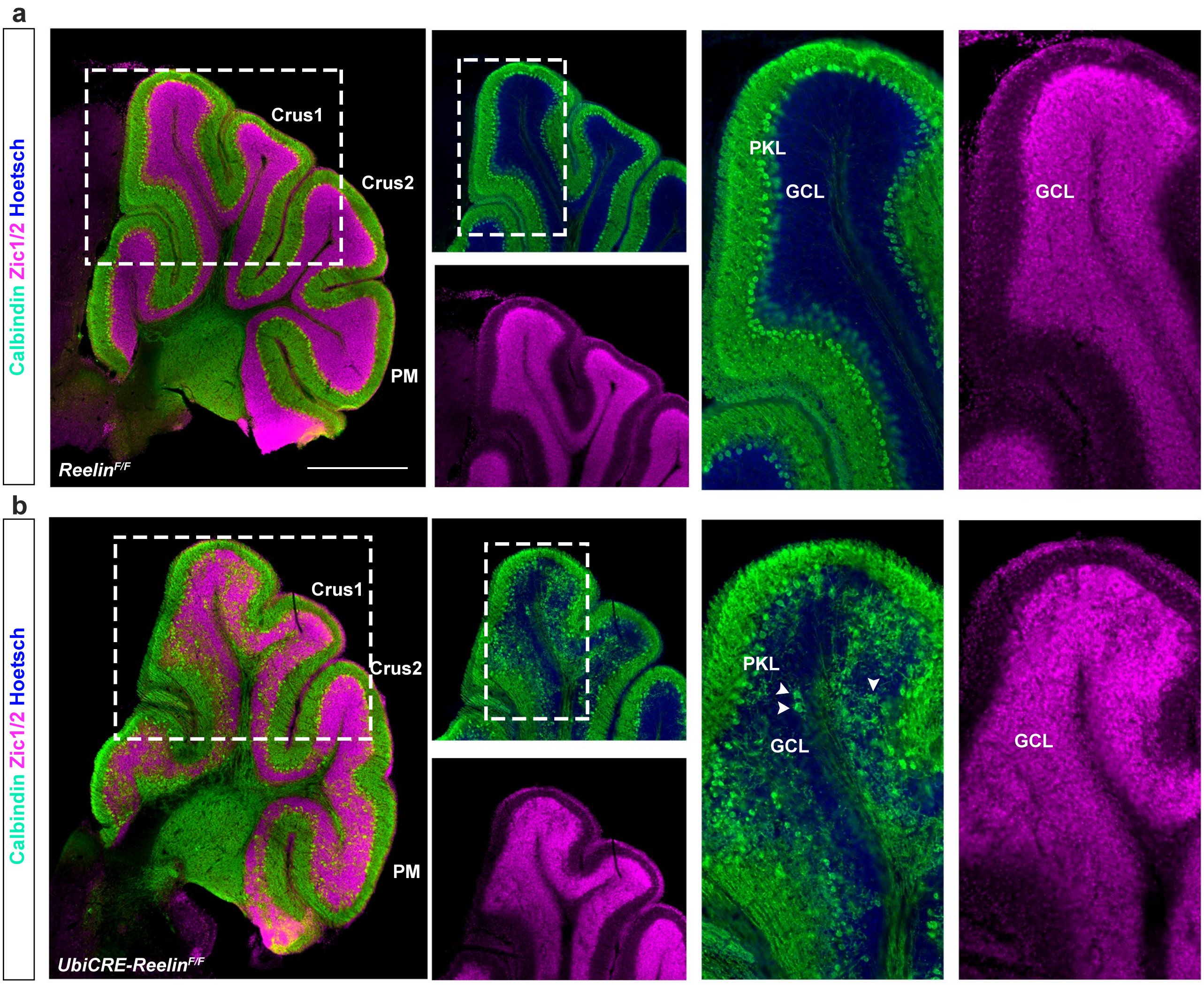

SUPPLEMENTARY FIG. 4

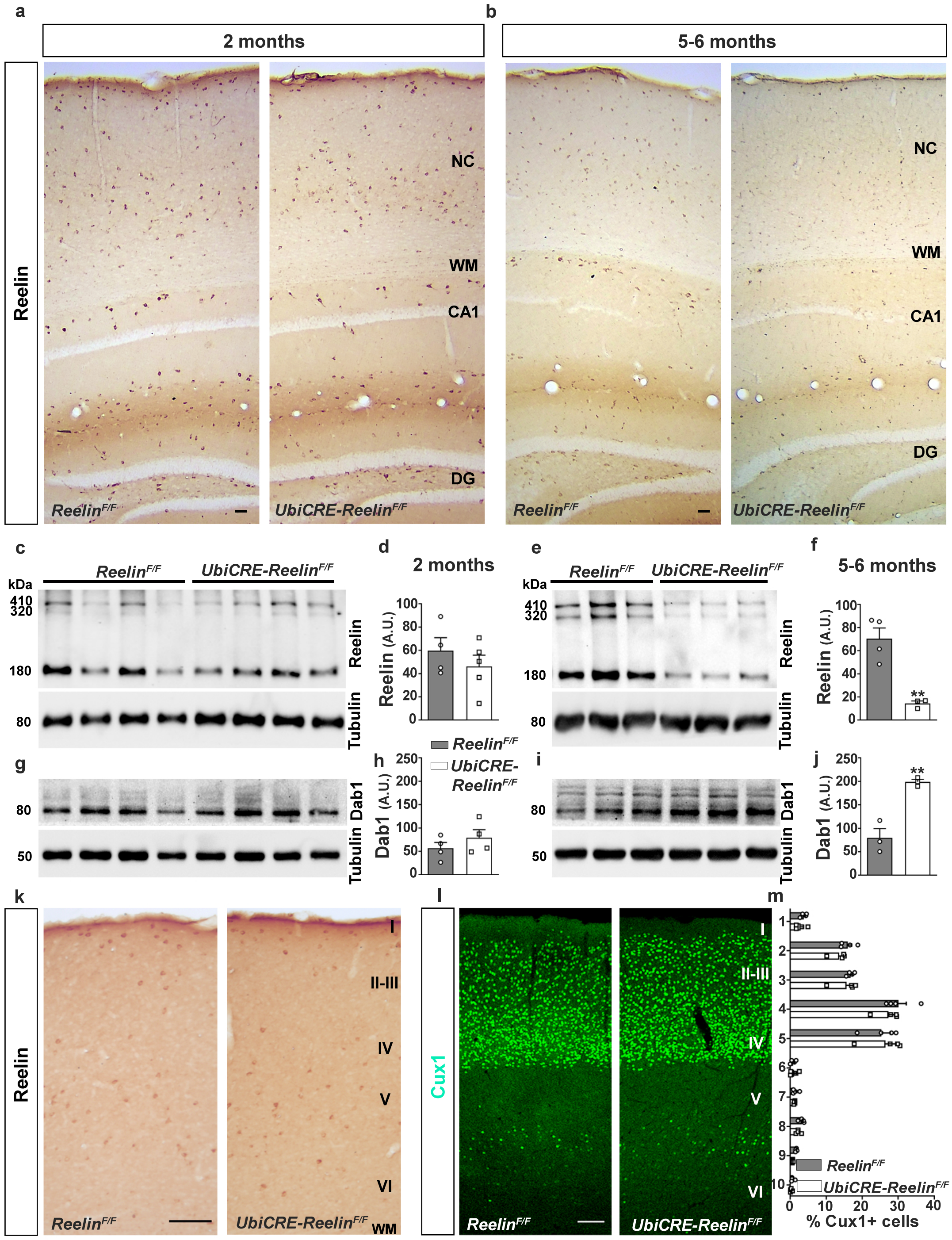

SUPPLEMENTARY FIG. 5

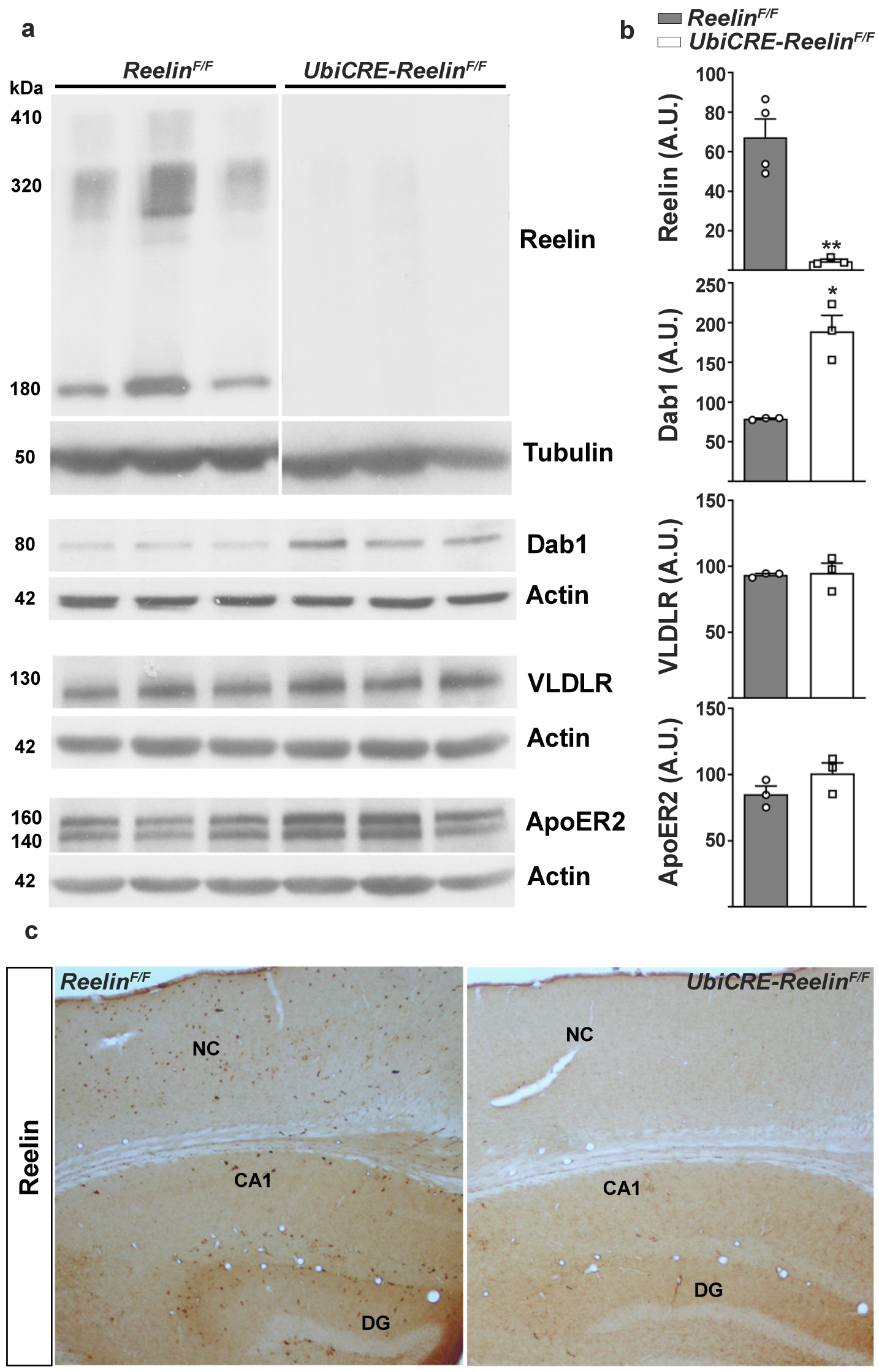

SUPPLEMENTARY FIG. 6

a

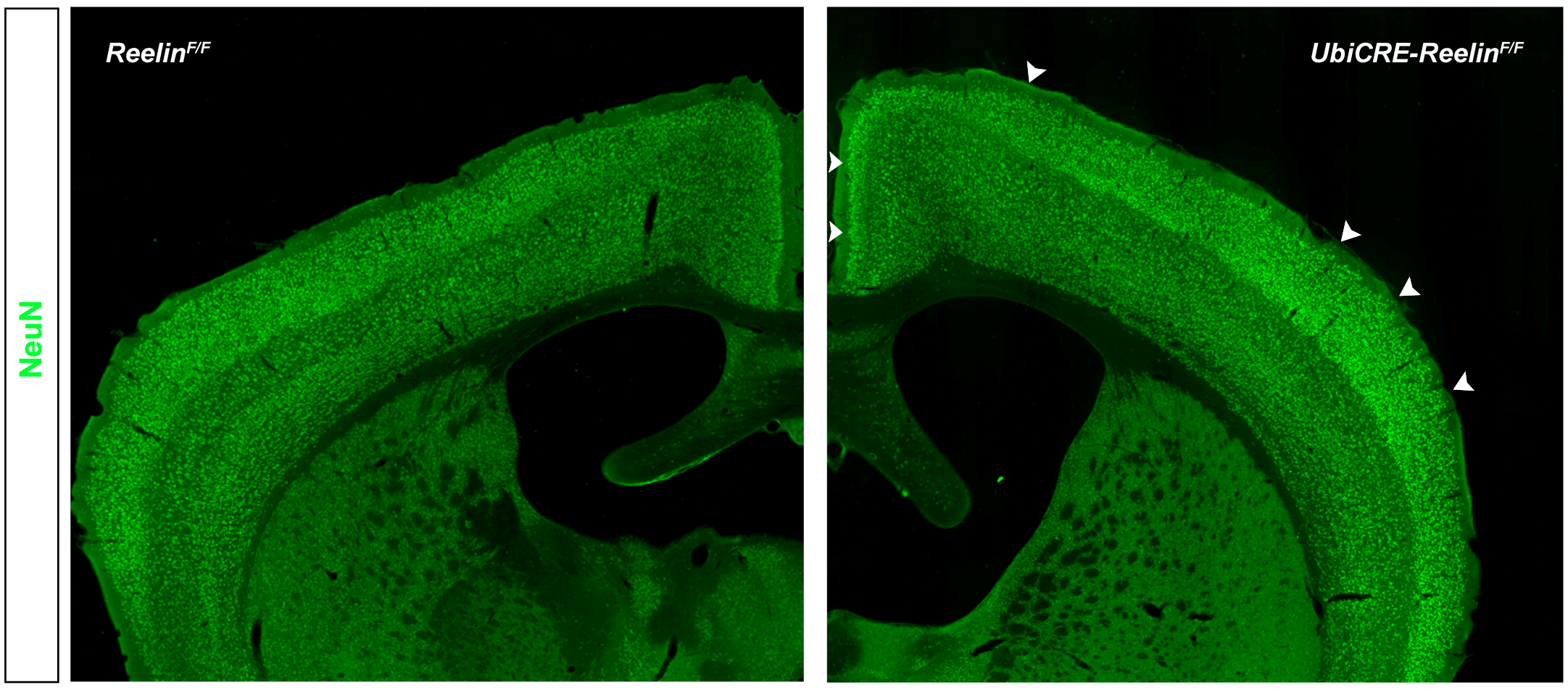

b

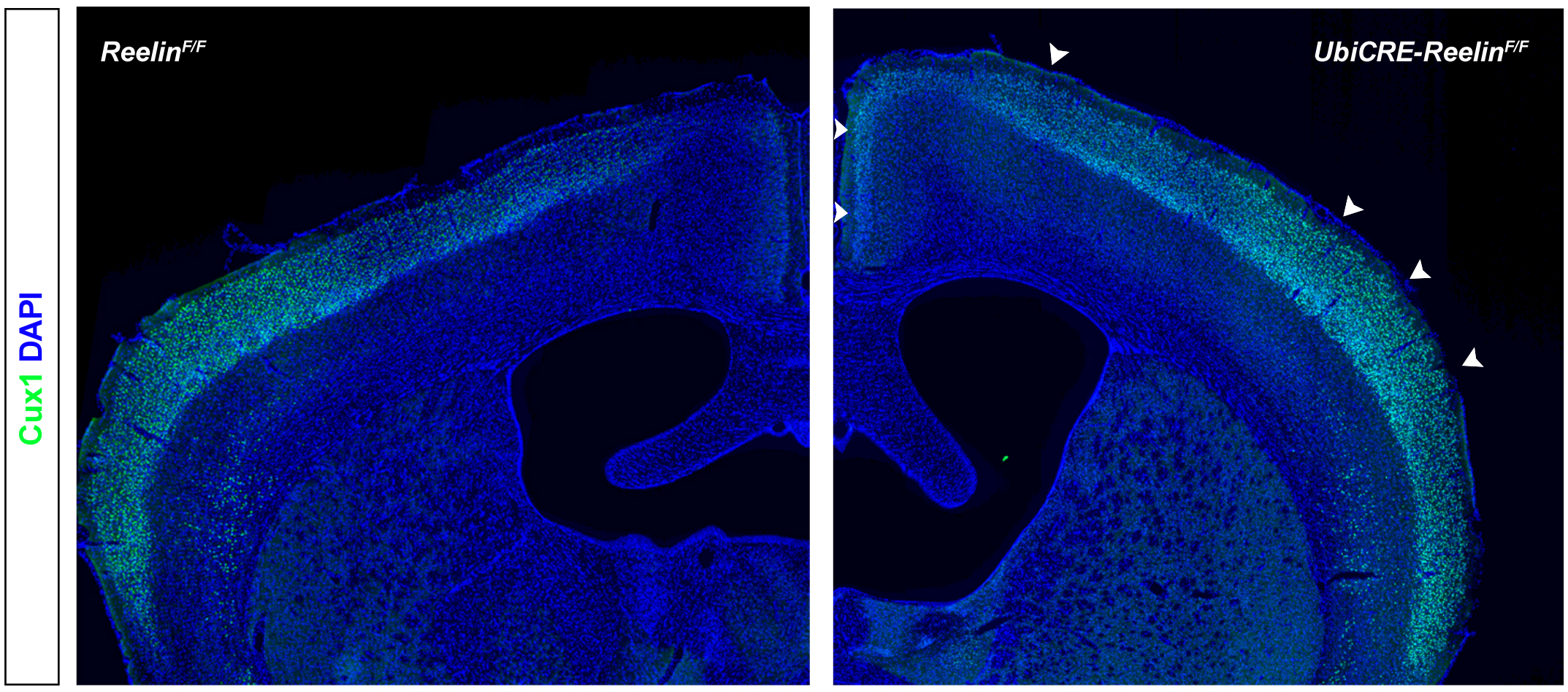

SUPPLEMENTARY FIG. 7

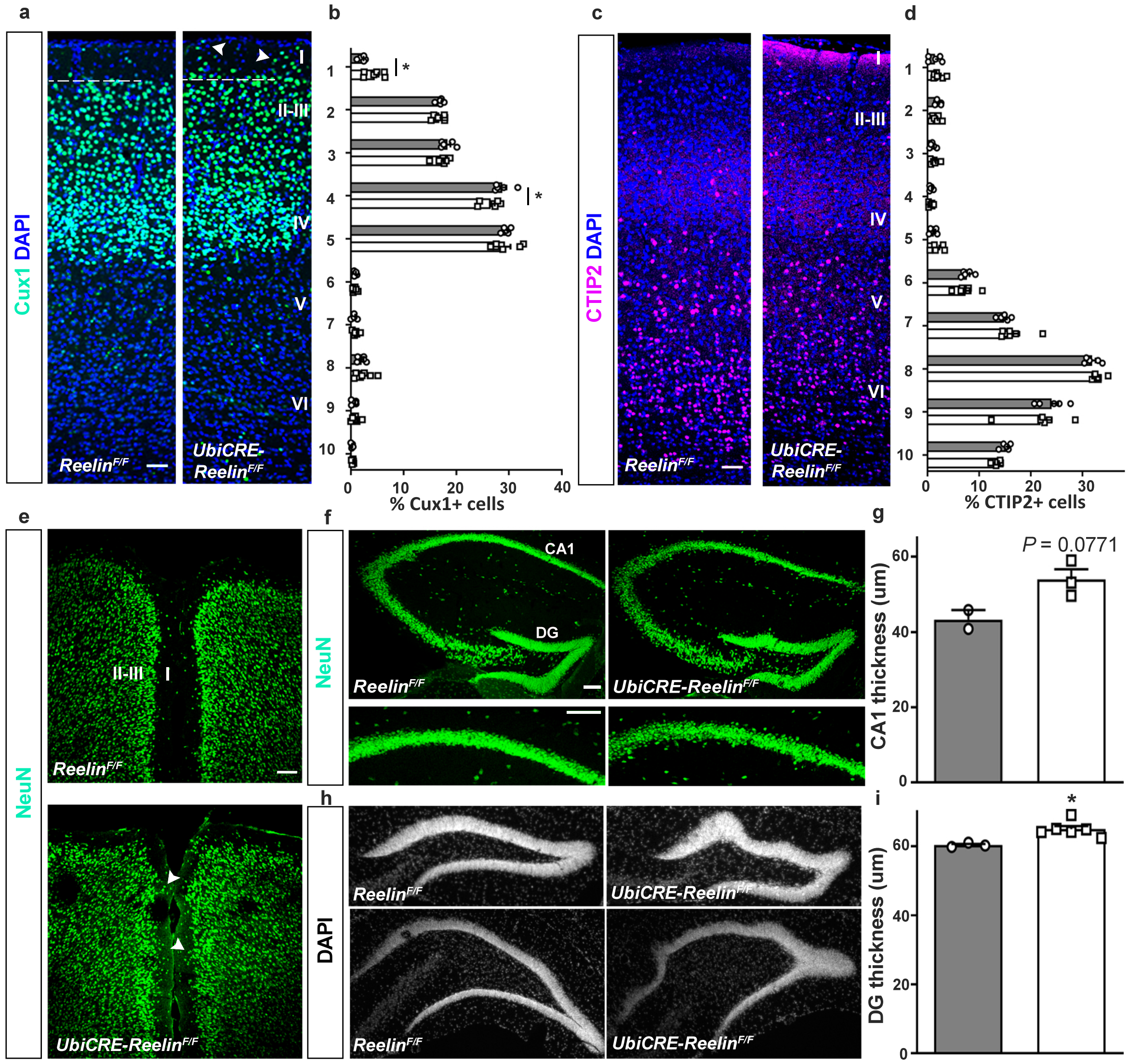

SUPPLEMENTARY FIG. 8

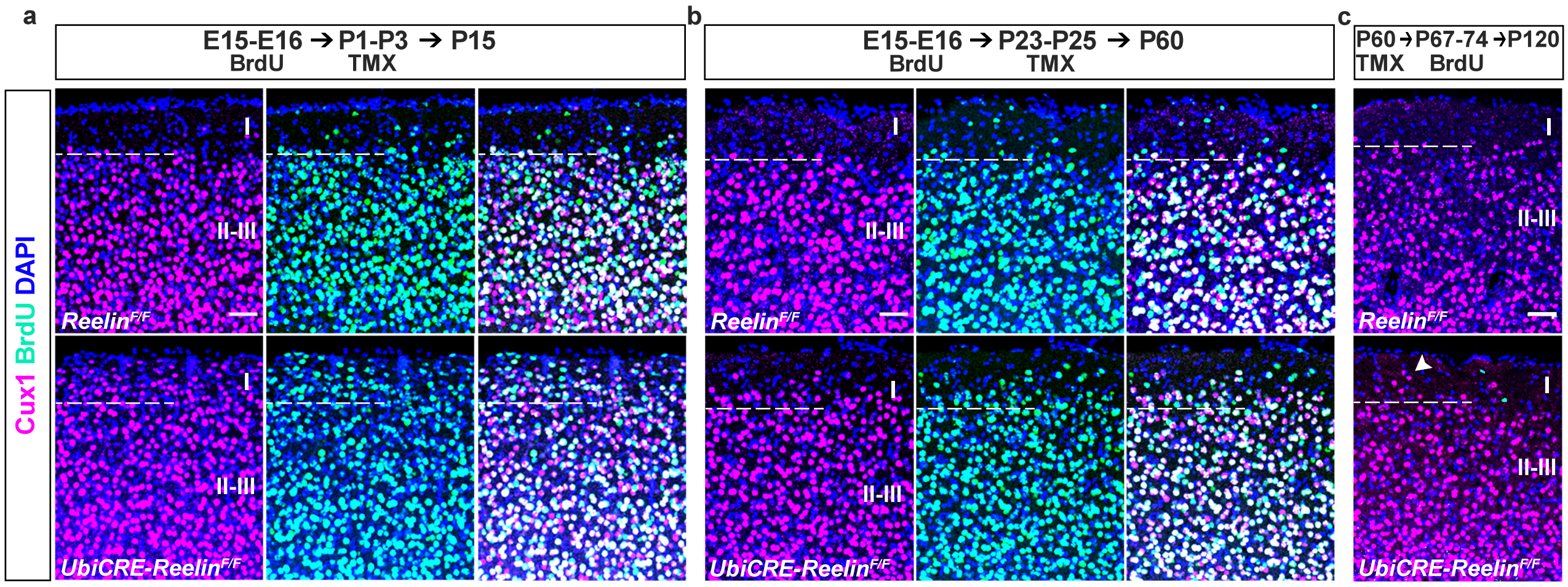

SUPPLEMENTARY FIG. 9

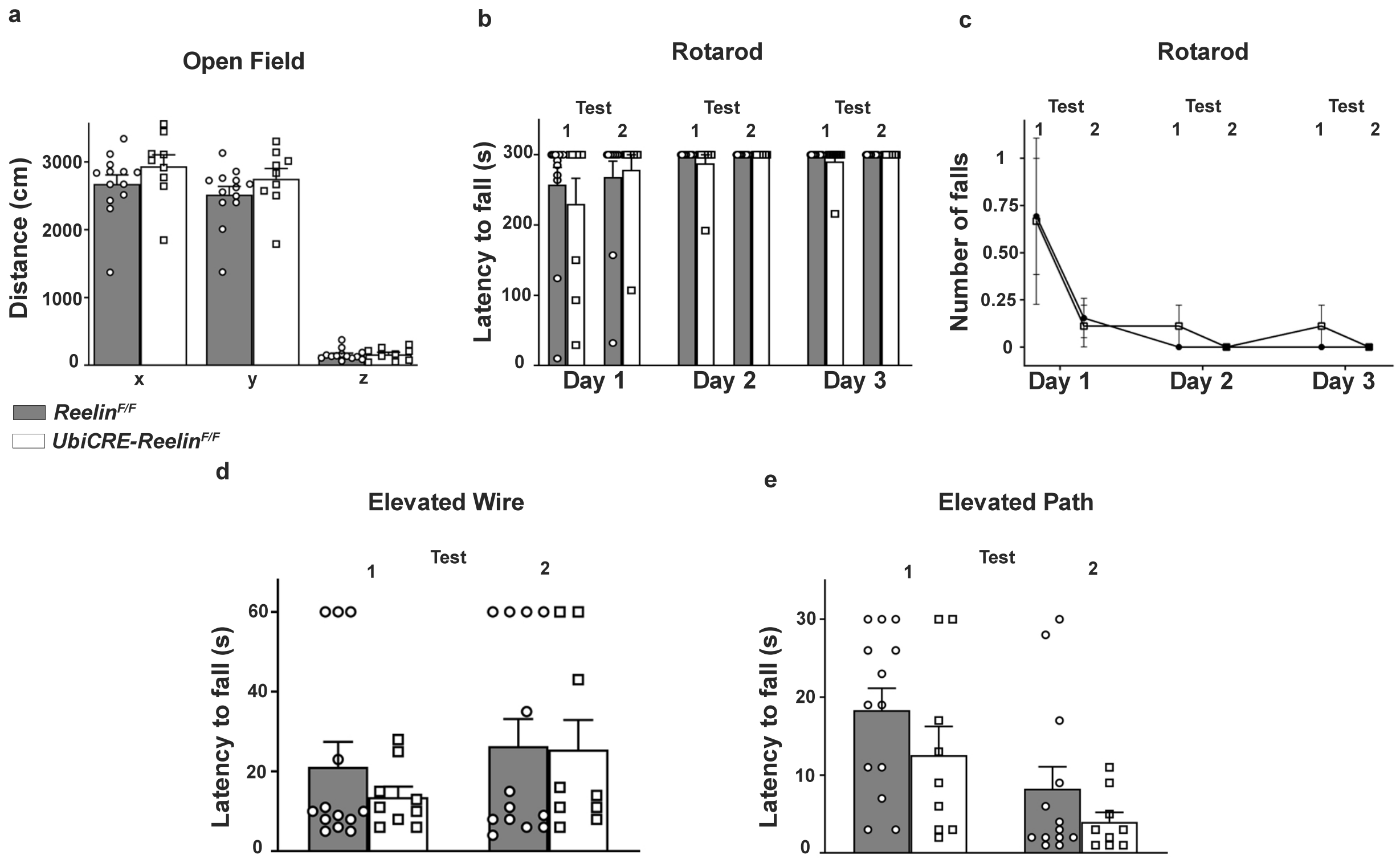

Supplementary Fig. 1: *UbiCRE-Reelin$^{F/F}$* line generation and inactivation at early postnatal stages.

a, To generate the floxed *Reelin* line (*Reelin$^{F/F}$*), LoxP sites were introduced flanking the exon 1 of the *Reln* gene. The line was bred to homozygosity and then crossed with the *Ubi-CreER^{T2}* line to obtain the *UbiCRE-Reelin$^{F/F}$* line, which expresses a tamoxifen-inducible Cre recombinase under the control of the ubiquitin promoter. **b**, Total brain homogenate western blot (excluding cerebellum) against Reelin before (P0), during (P2-3) and after (P4, P6) tamoxifen administration to P1-3 pups. Data evidence a significant loss of Reelin protein by P3-4, and its absence by P6, in *UbiCRE-Reelin$^{F/F}$* mice. **c**, Representative image of an immunohistochemistry against Reelin in P15 control (*Reelin$^{F/F}$*, left) and Reelin-deficient (*UbiCRE-Reelin$^{F/F}$*, right) mice, further confirming the absence of Reelin in *UbiCRE-Reelin$^{F/F}$* mice. Scale bar, **c**, 200 µm. Neocortex (NC), cornus ammonis (CA), dentate gyrus (DG).

ALT TEXT: Genetic scheme, western blots, and brain staining illustrate generation of the conditional Reelin-inactivation model and the progressive loss of Reelin following early postnatal tamoxifen administration.

Supplementary Fig. 2: *Reln* inactivation at early postnatal stages specifically affects late-born cortical neurons.

a, **c**, Representative confocal images of inner (Tbr1+) and lower (CTIP2+) cortical layers of P15 control (*Reelin$^{F/F}$*) and Reelin-deficient mice (*UbiCRE-Reelin$^{F/F}$*), showing no differences in neuronal distribution between genotypes. **b**, Quantification of the percentage of Tbr1+ cells per bin. **d, e**, Representative image composition showing NeuN+ (**d**, mature neurons) and Cux1+ (**e**, upper layers) cell distribution in P15 *Reelin$^{F/F}$* and *UbiCRE-Reelin$^{F/F}$* mice. Note the striking and extensive invasion of layer I by upper-layer neurons (arrowheads) in the *UbiCRE-Reelin$^{F/F}$* model (right). Data represent mean ± s.e.m. Statistical analysis: two-way ANOVA with Bonferroni post hoc test, **b**, bin x genotype Interaction $p = 0.6144$, $F(9, 70) = 0.9579$; n.s. n = 4-5 mice per genotype. Scale bar, **a, c**, 100 µm. Cortical layers I to VI (I, II-III, IV, V, VI), neocortex (NC), hippocampus (H), thalamus (T), caudate putamen (CPu).

ALT TEXT: Cortical fluorescence images and a cell-distribution graph compare postnatal mice with floxed Reelin alleles and mice with UbiCRE-mediated Reelin inactivation. Inner- and lower-layer neurons show similar distributions, whereas upper-layer neurons extend abnormally into cortical layer I after Reelin inactivation.

Supplementary Fig. 3: Disrupted Purkinje cell positioning after *Reln* inactivation at early postnatal stages.

a, b, Representative confocal images of Purkinje cells (Calbindin+) and granule cells (Zic1/2+) in the lateral cerebellum of P15 *Reelin*$^{F/F}$ and *UbiCRE-Reelin*$^{F/F}$ mice after *Reln* inactivation at P1-P3. Note the more severe disorganization of Purkinje cells in the lateral folia of the *UbiCRE-Reelin*$^{F/F}$ model. Arrows indicate ectopic PKc. Scale bar, 700 µm. Crus of the ansiform lobule (Crus), Paramedian lobule (PM), Granular Cell Layer (GCL) and Purkinje Cell Layer (PKL).

ALT TEXT: Cerebellar fluorescence images compare postnatal mice with floxed Reelin alleles and mice with UbiCRE-mediated Reelin inactivation, showing marked displacement and disorganization of Purkinje cells in the lateral cerebellar folia after Reelin inactivation.

Supplementary Fig. 4: Cre-mediated recombination in the absence of TMX (leaking) precludes from using non-treated *UbiCRE-Reelin*$^{F/F}$ mice as controls.

a-b, Representative immunohistochemistry against Reelin, at 2 (**a**) and 5-6 months (**b**) in control (*Reelin*$^{F/F}$) and non-treated *UbiCRE-Reelin*$^{F/F}$ mice. At 2 months of age, mice present similar levels of Reelin in the brain whilst at 5-6 months there is a clear reduction in Reelin levels in non-treated *UbiCRE-Reelin*$^{F/F}$ compared to *Reelin*$^{F/F}$ mice. **c-j**, Brain homogenates WB analysis at 2 (**c-d, g-h**) and 5-6 months (**e-f, i-j**) showing similar amounts of Reelin and Dab1 in the brains of *Reelin*$^{F/F}$ and non-treated *UbiCRE-Reelin*$^{F/F}$ mice at 2 months and a substantial decrease in the levels of Reelin and concomitant increase in Dab1 levels in non-treated *UbiCRE-Reelin*$^{F/F}$ vs *Reelin*$^{F/F}$ mice at 5-6 months. These data suggests that cre-mediated recombination in the absence of TMX is occurring in *UbiCRE-Reelin*$^{F/F}$ mice and precludes from its use as controls. **k**, Reelin IHC at 2 months showing similar Reelin levels between *Reelin*$^{F/F}$ and non-treated *UbiCRE-Reelin*$^{F/F}$ mice. **l**, Representative confocal images illustrating the cortical distribution of Cux1+ cells in the somatosensory cortices of 2 months old adult *Reelin*$^{F/F}$ and non-treated *UbiCRE-Reelin*$^{F/F}$ mice. **m**, Percentage of Cux1+ cells per bin in the somatosensory cortex evidencing no significant differences between *Reelin*$^{F/F}$ and non-treated *UbiCRE-Reelin*$^{F/F}$ mice at this age. Data represent mean ± s.e.m. Statistical analysis: unpaired two-tailed Student's t-test, **d** , $t = 0.9302$ $df = 7$ $P = 0.3832$; **f** , $t = 5.014$ $df = 5$ $P = 0.0041$; **h** , $t = 1.068$ $df = 6$ $P = 0.3267$; **j** , $t = 5.886$ $df = 4$ $P = 0.0042$; two-way ANOVA with Bonferroni post hoc test, **m**, bin x genotype Interaction $p = 0.9129$, $F(9, 60) = 0.4313$; n.s. **, $P < 0.01$ n = 3-5 mice per genotype. Scale bar **a-b,k-l**, 100 µm. Cortical layers I to VI (I, II-III, IV, V, VI), white matter (WM), neocortex (NC), cornus ammonis (CA), dentate gyrus (DG).

ALT TEXT: Brain staining, western blots, and cortical distribution graphs compare mice with floxed Reelin alleles and untreated UbiCRE Reelin mice at 2 and 5 to 6 months. Reelin expression is similar at the earlier age but reduced in older untreated UbiCRE mice.

Supplementary Fig. 5: Inactivation of the *Reln gene* during adulthood.

a, Representative total brain homogenate western blot (excluding cerebellum) against Reelin, Dab1, VLDLR and ApoER2 from brains (5-6 month old) of *Reelin*$^{F/F}$ control and *UbiCRE-Reelin*$^{F/F}$ mice whose *Reln* gene was inactivated in adulthood (P60). **b**, Western blot quantifications for Reelin, Dab1, VLDLR and ApoER2 showing the absence of Reelin in *UbiCRE-Reelin*$^{F/F}$ brains, with a concomitant increase in Dab1 levels and no changes in the receptor's levels. **c**, Representative immunohistochemistry against Reelin, further confirming the absence of Reelin after tamoxifen administration to *UbiCRE-Reelin*$^{F/F}$ adult mice; a typical distribution of Reelin+ neurons was seen in controls (*Reelin*$^{F/F}$). Data represent mean ± s.e.m. Statistical analyses: unpaired two-tailed Student's t-test with Welch correction, **b**, Reelin, t = 6.695 df = 3.068 P = 0.0063; Dab1, t = 5.395 df = 2.007 P = 0.0325; *, $P < 0.05$. **, $P < 0.01$, n = 3-4 animals per genotype. Neocortex (NC), cornus ammonis (CA), dentate gyrus (DG).

ALT TEXT: Western blots, quantitative graphs, and brain staining compare mice with floxed Reelin alleles and mice with UbiCRE-mediated Reelin inactivation during adulthood, showing loss of Reelin and increased Dab1 without visible changes in VLDLR or ApoER2 levels.

Supplementary Fig. 6: *Reln* inactivation at adult stages results in intermittent adult postmitotic neuronal dispersion into layer I.

a, b, Representative composited image showing NeuN+ (**a**, mature neurons) and Cux1+ (**b**, upper layers) cell distribution along the neocortex in adult *Reelin*$^{F/F}$ and *UbiCRE-Reelin*$^{F/F}$ mice (5-6 month-old) after *Reln* inactivation at P60. Note the intermittent and prominent invasion of layer I by upper-layer neurons (arrowheads) in the *UbiCRE-Reelin*$^{F/F}$ model.

ALT TEXT: Tile-scan fluorescence images compare adult mice with floxed Reelin alleles and mice with UbiCRE-mediated Reelin inactivation, showing intermittent clusters of upper-layer neurons extending into cortical layer I after Reelin inactivation.

Supplementary Fig. 7: Changes induced by *Reln* inactivation persist throughout adult life.

a, c, e, Representative confocal images illustrating the cortical distribution of Cux1+ (**a**), CTIP2+ (**c**) and NeuN+ (**e**) cells in the somatosensory (**a, c**) and the cingulate (**e**) cortices of old adult *Reelin*$^{F/F}$ and *UbiCRE-Reelin*$^{F/F}$ mice (11-12 months after tamoxifen administration at P60). **b, d**,

Percentage of Cux1+ (**b**) and CTIP2+ (**d**) cells per bin in the somatosensory cortex. The results further confirm the presence of intermittent clusters of upper-layer neurons invading layer I (arrowheads) in *UbiCRE-Reelin*$^{F/F}$ mice. **f,** NeuN immunostaining showing a wider pyramidal cell distribution in the *UbiCRE-Reelin*$^{F/F}$ hippocampus. **g,** Analysis of CA1 thickness. **h**, Representative DG images evidencing the persistence of granule cell dispersion and the presence of protruding areas in *UbiCRE-Reelin*$^{F/F}$ mice long after tamoxifen inactivation. **i**, Analysis of DG thickness. Data represent mean ± s.e.m. Statistical analyses: two-way ANOVA with Bonferroni post hoc test, **c**, bin x genotype Interaction F(9, 90) = 2.446 *P* = 0.0154; n = 5-6 mice per genotype, **d**, bin F(9, 90) = 403.9 *P* < 0.0001, n = 5-6 mice per genotype; unpaired two-tailed Student's t-test, **g**, t = 2.649 df = 3 *P* = 0.0771; n = 2-3 mice per genotype; **i**, t = 3.408 df = 7 *P* = 0.0113; n = 3-6 mice per genotype; *, *P* < 0.05. Scale bar, **a-b**, 50 µm, **e, f,** 100 µm. Cortical layers I to VI (I, II-III, IV, V, VI), cornus ammonis (CA), dentate gyrus (DG).

ALT TEXT: Cortical and hippocampal fluorescence images and quantitative graphs compare older mice with floxed Reelin alleles and mice with UbiCRE-mediated Reelin inactivation, showing persistent cortical neuronal displacement and broader hippocampal cell layers.

Supplementary Fig. 8: Late-born cortical neurons invade layer I after *Reln* inactivation.

a-c, Representative BrdU and Cux1 immunostaining confocal images of brains from: P15 mice injected with BrdU at E15-16 and *Reln* inactivated with tamoxifen at P1-3 (**a**), adult mice injected with BrdU at E15-16 and Reelin inactivated with tamoxifen at young adult stages (**b**), and adult mice *Reln* inactivated with TMX at adult stages and subsequently injected with BrdU for several days (**c**). Images clearly indicate that cells invading layer I after *Reln* inactivation correspond to late-born cortical neurons (presence of neurons doubly labeled with BrdU and Cux1 in **a** and **b**) and not to adult-born neurons (absence of doubly labeled neurons in **c**). Arrowhead indicates ectopic cells. Scale bar, **a-c**, 50 µm. Cortical layer I (I), layers II-III (II-III).

ALT TEXT: Brain birth-dating images from mice with floxed Reelin alleles and mice with UbiCRE-mediated Reelin inactivation compare embryonically and adult-labeled cortical neurons. Ectopic neurons in layer I colocalize with embryonic birth-dating labels but not with labels administered during adulthood.

Supplementary Fig. 9: *Reln* inactivation in the adult is not associated with motor impairments.

Mice that were *Reln* inactivated in adulthood were subjected to a battery of tests to assess their motor performance. **a**, Analysis of the distance travelled in the open field test. **b-c**, Analysis of the latency to fall and the number of falls in the rotarod test. **d-e**, Analysis of the latency to fall

from the elevated wire (**d**) and the elevated path (**e**) paradigms. Altogether, the tests indicate that *RELN* inactivation at adult stages does not alter motor performance. Data represent mean ± s.e.m. Statistical analyses: Reelin$^{F/F}$ vs. *UbiCRE-Reelin*$^{F/F}$, ANOVA *F*-test, or Kruskal-Wallis ANOVA on ranks (*H*-statistic). **a**, activity-x: H = 2.258 with 1 degrees of freedom, P = 0.133; activity-y: H = 2.062 with 1 degrees of freedom, P = 0.151; activity-z: $F(1,20)$ = 0.024, P = 0.879. **b** and **c**, tests 1 and 2; days 1, 2 and 3: for all the multiple comparisons $P > 0.228$. **d**, test 1: H = 0.0721 with 1 degrees of freedom, P = 0.788; test 2: H = 0.194 with 1 degrees of freedom, P = 0.660. **e**, test 1: $F(1,20)$ = 1.576, P = 0.224; test 2: H = 0.554 with 1 degrees of freedom, P = 0.45.

ALT TEXT: Behavioral graphs compare adult mice with floxed Reelin alleles and mice with UbiCRE-mediated Reelin inactivation in open-field, rotarod, elevated-wire, and elevated-path tests, with broadly overlapping motor performance between groups.
